# Modeling the protein binding non-linearity in population pharmacokinetic model of valproic acid in children with epilepsy: a systematic evaluation study

**DOI:** 10.3389/fphar.2023.1228641

**Published:** 2023-10-06

**Authors:** Lina Zhang, Maochang Liu, Weiwei Qin, Dandan Shi, Junjun Mao, Zeyun Li

**Affiliations:** ^1^ Department of Neurology, The First Affiliated Hospital of Zhengzhou University, Zhengzhou, China; ^2^ Department of Pharmacy, Wuhan Children’s Hospital, Tongji Medical College, Huazhong University of Science and Technology, Wuhan, Hubei, China; ^3^ Department of Pharmacy, Huashan Hospital, Fudan University, Shanghai, China; ^4^ Department of Pediatrics, The First Affiliated Hospital of Zhengzhou University, Zhengzhou, China; ^5^ Department of Pharmacy, The First Affiliated Hospital of Zhengzhou University, Zhengzhou, China

**Keywords:** population pharmacokinetics, valproic acid, external evaluation, pediatric epilepsy, protein-binding saturation, therapeutic drug monitoring

## Abstract

**Background:** Several studies have investigated the population pharmacokinetics (popPK) of valproic acid (VPA) in children with epilepsy. However, the predictive performance of these models in the extrapolation to other clinical environments has not been studied. Hence, this study evaluated the predictive abilities of pediatric popPK models of VPA and identified the potential effects of protein binding modeling strategies.

**Methods:** A dataset of 255 trough concentrations in 202 children with epilepsy was analyzed to assess the predictive performance of qualified models, following literature review. The evaluation of external predictive ability was conducted by prediction- and simulation-based diagnostics as well as Bayesian forecasting. Furthermore, five popPK models with different protein binding modeling strategies were developed to investigate the discrepancy among the one-binding site model, Langmuir equation, dose-dependent maximum effect model, linear non-saturable binding equation and the simple exponent model on model predictive ability.

**Results:** Ten popPK models were identified in the literature. Co-medication, body weight, daily dose, and age were the four most commonly involved covariates influencing VPA clearance. The model proposed by Serrano et al. showed the best performance with a median prediction error (MDPE) of 1.40%, median absolute prediction error (MAPE) of 17.38%, and percentages of PE within 20% (F_20_, 55.69%) and 30% (F_30_, 76.47%). However, all models performed inadequately in terms of the simulation-based normalized prediction distribution error, indicating unsatisfactory normality. Bayesian forecasting enhanced predictive performance, as *prior* observations were available. More *prior* observations are needed for model predictability to reach a stable state. The linear non-saturable binding equation had a higher predictive value than other protein binding models.

**Conclusion:** The predictive abilities of most popPK models of VPA in children with epilepsy were unsatisfactory. The linear non-saturable binding equation is more suitable for modeling non-linearity. Moreover, Bayesian forecasting with *prior* observations improved model fitness.

## 1 Introduction

Epilepsy is a common chronic neurological disorder in pediatrics that burdens patients with huge biological, psychological, and social hardship and requires long-term antiseizure medications (ASMs) therapy (Moshe et al., 2015; Patsalos et al., 2018). As a first-line ASM for both focal and generalized epilepsy syndromes and seizures in male children, the advantages of valproic acid (VPA) include a wide spectrum of antiepileptic properties and an acceptable tolerability profile (Johannessen Landmark and Svein, 2016; Romoli et al., 2019).

The oral bioavailability of VPA is almost complete in all commonly prescribed formulations (Romoli et al., 2019). VPA exhibits high (90%–95%), concentration-dependent, and saturable plasma protein-binding (Kodama et al., 1992a; Johannessen and Johannessen, 2003), resulting in non-linear pharmacokinetics (PK) (Zaccara et al., 1988). VPA is mainly metabolized through glucuronidation via uridine diphosphate glucuronosyltransferase (UGT), β-oxidation in the mitochondria, and cytochrome P450 (CYP)-catalyzed oxidation in the liver. Ultimately, the metabolites are mainly excreted in the urine (Silva et al., 2008; Romoli et al., 2019).

In clinical practice, the clearance (CL/*F*) and plasma concentrations of VPA vary greatly among individuals, which can be primarily ascribed to demographic and clinical characteristics, concomitant medications, and genetic variants (Ghodke-Puranik et al., 2013; Zhu et al., 2017). Owing to the narrow therapeutic range (50–100 mg/L) and considerable interpatient PK variability of VPA, close therapeutic drug monitoring (TDM) and individualized medication are indispensable (Patsalos et al., 2008; Johannessen Landmark et al., 2020).

Individual PK parameters required for optimizing dosage regimens can be obtained using population pharmacokinetic (popPK) modeling combined with Bayesian forecasting (Sheiner et al., 1979). Unlike traditional PK approaches, popPK analysis is superior in estimating intra- and inter-subject variabilities and predicting plasma concentrations by quantifying the effects of relevant covariates on PK parameters (Sun et al., 1999; Ette and Williams, 2004). This method allows the use of sparse TDM data and is suitable for studies in children due to logistical and ethical constraints with respect to performing intensive sampling strategies in this vulnerable population.

Several popPK models have been established to quantitatively explore the PK characteristics of VPA (Methaneethorn, 2018; Zang et al., 2022). Body weight, sex, age, VPA daily dose, and concomitant medications have been most frequently reported as covariates that influence VPA CL/*F*. Approximately one-third of the studies have assessed the predictive performances of the final models. The predictive abilities of VPA popPK models for patients with bipolar disorder in China (Zang et al., 2022) and patients with mania in Thailand (Methaneethorn and Leelakanok, 2021) have been externally evaluated. However, popPK models and the influence of incorporating non-linear properties on the model’s predictive ability for children with epilepsy have not been evaluated. The modeling strategy and functional forms of the covariates involved may affect the model’s predictive ability (Mao et al., 2020).

VPA concentrations are disproportionately higher when protein binding is saturated, particularly as the concentrations of VPA exceed 50 mg/L (Winter, 2010). To investigate the non-linear relationship of VPA concentrations between the total and free serum, a one-binding site model (Dutta et al., 2007), Langmuir equation (Ueshima et al., 2008), dose-dependent maximum effect model (Ding et al., 2015), linear non-saturable binding equation (Gu et al., 2021), and the VPA daily dose incorporated as a covariate have been used in modeling development. However, most published models have been developed using empirical covariate selection and have not considered non-linear properties. Theoretical covariate selection, which is based on the understanding of PK mechanisms rather than solely on the data, combined with the relationships between covariates and parameters, may help improve model prediction (Danhof et al., 2008; Mao et al., 2020).

To fully exploit the benefits of model-informed precision dosing, the most suitable popPK model that can accurately describe the PK process in the target population should be obtained (Kluwe et al., 2020). This study aimed to systematically assess the predictive performance of published popPK models for VPA in children with epilepsy as well as explore the potential effects of protein binding modeling strategies on model transferability.

## 2 Materials and methods

### 2.1 Review of studies on popPK analyses of VPA in children with epilepsy

A systematic review of studies on the popPK of VPA in children with epilepsy was conducted by retrieving literature from PubMed, Web of Science, and Embase from inception up to 30 June 2022. The criteria for inclusion in published models were as follows: (1) a study using a non-linear mixed effect modeling approach to analyze VPA PK parameters in children with epilepsy, and (2) publications written in English. Studies were excluded if (1) they were methodological papers or reviews, (2) the required information was insufficient for external evaluation, and (3) the data or cohorts overlapped. In addition, popPK studies, including genetic polymorphisms as covariates, were excluded as genotyping is not routinely performed in TDM of VPA. Furthermore, citations in the identified publications were screened.

### 2.2 Data for external evaluation

#### 2.2.1 Participants

Overall, 202 Chinese children with epilepsy (139 males and 63 females) undergoing VPA treatment and who had undergone routine monitoring of plasma concentrations at Wuhan Children’s Hospital between January 2016 and November 2018 were eligible. The clinical and demographic characteristics of each participant, including sex, age, body weight (BW), dosage regimen, concomitant drugs, and seizure control, were obtained at each TDM visit for the current evaluation. Patients with missing information on the required covariates, hepatic or renal impairment, abnormal albumin levels, or poor medication adherence were excluded. The Ethics Committee of Wuhan Children’s Hospital approved the protocol (Serial Number: 2015015), and patients’ direct relatives were well informed and signed informed consent declarations voluntarily.

#### 2.2.2 VPA concentration determination

VPA total plasma concentrations were measured by gas chromatography (GC). The calibration range of the hydrogen flame ionization detector was 12.5–150 mg/L. The detection limit was 1 mg/L, and the coefficient of variation was below 10%, as reported in an earlier study (Liu et al., 2020).

### 2.3 External predictive ability evaluation

The assessment of predictive ability was conducted with NONMEM^®^ 7.4 (ICON Development Solutions, Ellicott City, MD, United States) and assisted by Intel Fortran XE 2011 Update 13 (Intel Corp, Santa Clara, CA, United States). We reconstructed published popPK models by incorporating reported parameters and subsequently assessed the predictive performance of the eligible models using prediction- and simulation-based diagnostics, as well as Bayesian forecasting (Zhao et al., 2016a; Mao et al., 2018), with an external dataset. To analyze the output of NONMEM, R software (version 4.2.1, http://www.r-project.org/) was used.

#### 2.3.1 Prediction-based diagnostics

For each participant, we calculated the prediction error (PE%) using population predictions (PREDs) and corresponding observations (OBS) according to Eq. 1. To evaluate the predictive accuracy and precision, median prediction error (MDPE) and median absolute prediction error (MAPE) were used (Sheiner and Beal, 1981).

$$\text{PE }\left(\%\right)=\left(\frac{\text{PRED}-\text{OBS}}{\text{OBS}}\right)\times 100$$ (1)

Subsequently, we calculated the PE% within ±20% (F_20_) and ±30% (F_30_) to serve as an indicator of the combined performance of model accuracy and precision. The candidate model’s predictive ability was considered to be satisfactory if the following criteria were met: MDPE ≤ ± 15%, MAPE ≤30%, F_20_ > 35%, and F_30_ > 50% (Mao et al., 2018). The PE% was visualized using boxplots and cumulative distribution curve plots.

#### 2.3.2 Simulation-based diagnostics

The evaluation and simulated data were statistically compared via a prediction-corrected visual predictive check (pcVPC) (Bergstrand et al., 2011) and normalized prediction distribution error (NPDE) (Comets et al., 2008) to assess each candidate model’s predictive ability for VPA via simulation. Based on the reported final model parameters, 2,000 times simulations of the dataset were carried out.

The graphical visualizations and calculations for pcVPC were conducted using PsN. The comparisons between the simulations and the observations at different time points were performed in pcVPCs using automatic binning. The NPDE was determined using an R package (NPDE, version 2.0, www.npde.biostat.fr) (Comets et al., 2008). To test the normal distribution property of NPDE data, diagnostic graphs were generated. Statistical tests were conducted with the null hypothesis, and the derived model satisfactorily accounted for the evaluation data. The hypothesis was examined with the Wilcoxon signed-rank test, Fisher’s exact variance test, and Shapiro-Wilk test, as appropriate (Brendel et al., 2010).

#### 2.3.3 Bayesian forecasting

To assess the impact of priors on model predictive performance, maximum *a posteriori* Bayesian (MAPB) forecasting using data from individuals with observed concentrations was performed. For each patient, one *prior* measurement was used to predict the individual prediction (IPRED) of the last observation, and the individual PE% (IPE%) was calculated using Eq. 2 to assess the model’s accuracy. Further details are shown in Supplementary Text S1.

$$\text{IPE }\left(\%\right)=\left(\frac{\text{IPRED}-\text{OBS}}{\text{OBS}}\right)\times 100$$ (2)

The median IPE% (MDIPE), median absolute IPE% (MAIPE), and IF_20_ and IF_30_ (representing F_20_ and F_30_ of IPE%, respectively) were calculated to assess the model’s predictive performance with increasing prior information.

### 2.4 The impact of protein binding modeling strategy

VPA is known to follow non-linear PK based on concentration-dependent protein binding (Zaccara et al., 1988). Based on the review of literature and data characteristics, a one-compartment model with first-order absorption was used as base model to describe VPA PK. Subsequently, five protein-binding modeling strategies (Eqs 3–7) were applied to compare the potential effect of functional forms and the non-linearity of covariates on the model’s predictive performance.

*Model I*: One-binding site model (Eq. 3)

$$C_{b}=\frac{N\times K\times C_{u}\times \text{ALB}}{1+K\times C_{u}}$$ (3)

where C_b_ and C_u_ represent the bound and unbound plasma concentrations of VPA, respectively, and ALB is the albumin level. The number of binding sites (N) per unit of single-site binding material was set as 1.98 while the binding site association constant (K) was set to 15.5 mM^−1^ as reported (Dutta et al., 2007).

*Model II*: Langmuir equation (Eq. 4)

$$C_{b}=\frac{B_{m}\times C_{u}}{K_{d}+C_{u}}$$ (4)

The dissociation constant of VPA (K_d_) and maximum binding site concentration (B_m_) were set to 7.8 and 130 mg/L, respectively (Ueshima et al., 2008).

*Model III*: Dose-dependent maximum effect model (Eq. 5)

$$\text{CL}/F={CL}_{P}\times \left(1+\frac{E_{\max}\times {DD}^{\gamma }}{{{DD}_{50}}^{\gamma }+{DD}^{\gamma }}\right)$$ (5)

where CL_p_ is the apparent plasma clearance, E_max_ is the maximum effect of VPA, DD_50_ is the daily dose (mg kg^−1^ day^−1^) when E_max_ is increased by 50%, and the sigmoid decline slope is specified by the Hill coefficient (γ). E_max_ and γ were set to 2.8 and 1.68, respectively, as reported (Ding et al., 2015).

*Model IV*: Linear non-saturable binding equation (Eq. 6)

$$C_{b}=\frac{B_{m}\times C_{u}}{K_{d}+C_{u}}+NS\times C_{u}$$ (6)

where NS is the slope of non-saturable protein-binding. K_d_, B_m_, and NS were set to 2.12, 67.3 mg/L, and 2.25, respectively (Gu et al., 2021).

*Model V*: The simple exponent model (Eq. 7)

$$CL/F={CL}_{P}\times {\left(\frac{DD}{25}\right)}^{k}$$ (7)

where k is the exponent of the daily dose, which was estimated using our dataset.

The evaluation approaches comprise the aforementioned diagnostic methods based on prediction and simulation as well as Bayesian forecasting methods. The tendency plots based on established models Ⅲ and Ⅴ were used to assess the relation of the daily dose to CL/*F* within these two modeling strategies.

## 3 Results

### 3.1 Review of published popPK analyses on VPA in children with epilepsy

**FIGURE 1:**
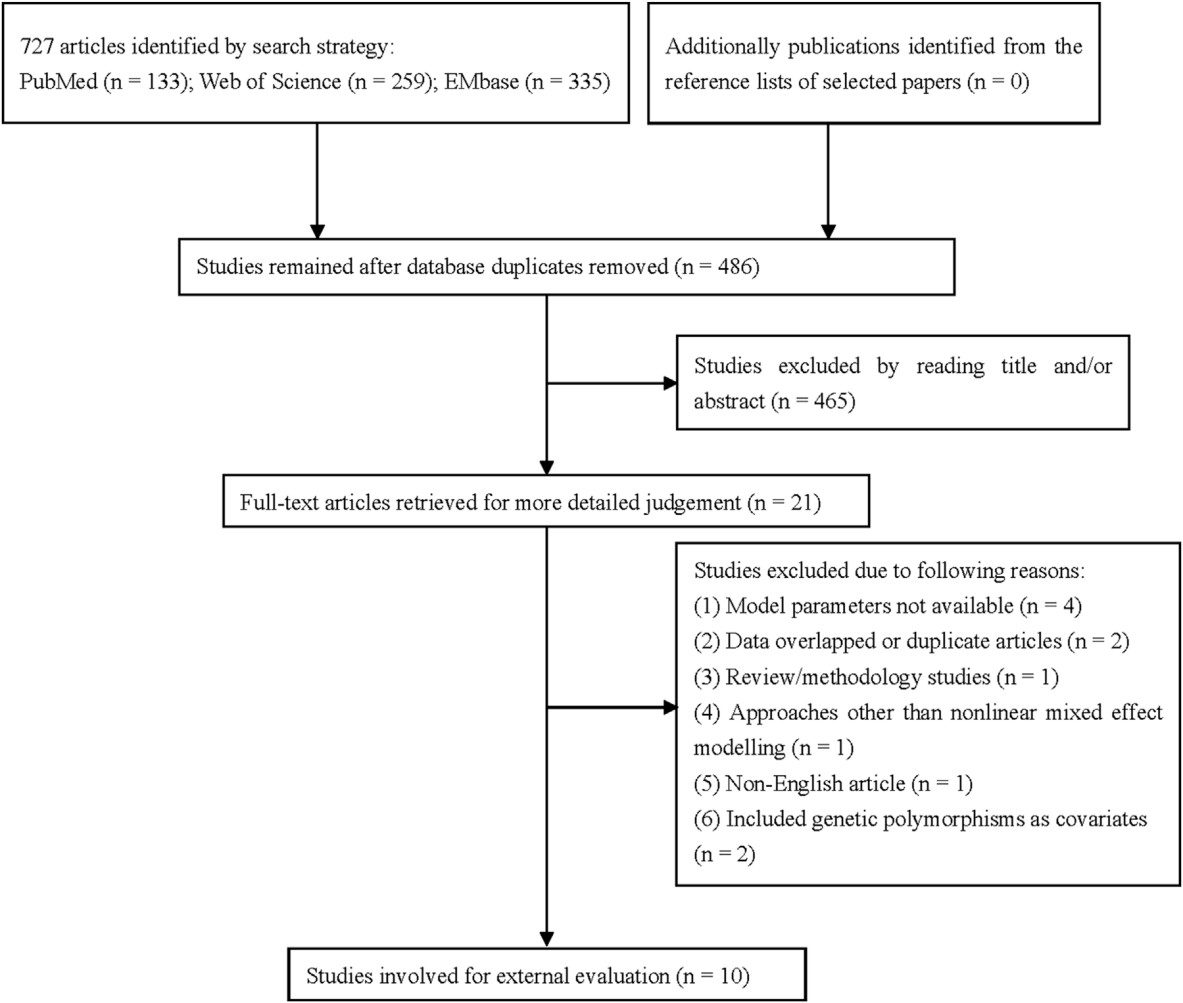
Overview of the literature search strategy. *n*, number of articles returned by search.

After literature retrieval (Figure 1; Supplementary Text S2), 10 published popPK studies on VPA in children with epilepsy were identified for external evaluation (Serrano et al., 1999; Desoky et al., 2004; Jiang et al., 2007; Correa et al., 2008; Williams et al., 2012; Ogusu et al., 2014; Ding et al., 2015; Rodrigues et al., 2018; Gu et al., 2021; Teixeira-da-Silva et al., 2022). Four studies were conducted in East Asian countries [three in China (Jiang et al., 2007; Ding et al., 2015; Gu et al., 2021) and one in Japan (Ogusu et al., 2014)], three in Europe [two in Spain (Serrano et al., 1999; Teixeira-da-Silva et al., 2022) and one in France (Rodrigues et al., 2018)], two in North America [one each in Mexico (Correa et al., 2008) and United States (Williams et al., 2012)], and one in Africa (Desoky et al., 2004). Among these studies, four were multi-center studies (Jiang et al., 2007; Williams et al., 2012; Ding et al., 2015; Rodrigues et al., 2018) and the others were single-center studies. Additionally, three studies used sample size of ≤100 participants (Desoky et al., 2004; Williams et al., 2012; Rodrigues et al., 2018).

Four bioassays were used in specific publications. Specifically, fluorescence polarization immunoassays (FPIA) were used in seven studies (Serrano et al., 1999; Desoky et al., 2004; Jiang et al., 2007; Williams et al., 2012; Ding et al., 2015; Rodrigues et al., 2018; Teixeira-da-Silva et al., 2022), enzyme multiplied immunoassay technique (EMIT), cloned enzyme donor immunoassays (CEDIAs), and GC were used in the remaining three studies (Correa et al., 2008; Ogusu et al., 2014; Gu et al., 2021) (Table 1). The demographic and pharmacokinetic characteristics of the models are listed in Supplementary Table S1.

**TABLE 1:** Summary of published population pharmacokinetic studies of VPA in children with epilepsy.

Study (publication year)	Country (single/multiple sites)	Number of patients (male/female)	Total samples	VPA assay	Structural model	PK parameters and formula		BSV (%)	Residual error
Serrano et al. (1999)	Spain (single)	255 (128/127)	770	FPIA	1CMT	CL/*F*	0.012 × WT^0.715^ × (DDW)^0.306^ × (1.359, if concomitant CBZ)	21.4	15.6 mg/L
						V/*F*	0.24 × WT	/	
						K_a_	1.9 (fixed)	/	
Desoky et al. (2004)	Egyptian (single)	81 (52/29)	81	FPIA	1CMT	CL/*F*	0.105 + 0.000248 × DD + 0.0968 × AGE/20 + (0.151, if concomitant CBZ) + (0.0803, if uncontrolled epilepsy)	23.6	5.24 mg/L
						V/*F*	11.5 (fixed)	—	
						K_a_	4 for syrup, 1 for EC-tablet (fixed)	—	
Jiang et al. (2007)	China (multiple)	317 (195/122)	624	FPIA	1CMT	CL/*F*	0.106^(0.98 × CO)^ + 0.0157 × AGE	25.1	13.2 mg/L
							CO = 1 when co-medication exists		
						V/*F*	2.88 + 0.157 × WT	49.1	
						K_a_	0.251 + 2.24 (1 − HS), HS = 1 for SR-tablet, otherwise HS = 0	11.0	
Correa et al. (2008)	Mexico (single)	110 (63/47)	119	CEDIA	1CMT	CL/*F*	(0.0466 + 0.00363 × WT + 0.000282 × DD) × (1.236, if concomitant PB)	14.1	17.3 mg/L
						V/*F*	0.24 × WT	—	
						K_a_	1.2 (fixed)	—	
Williams et al. (2012)	United States (multiple)	52 (36/16)	231	FPIA	2CMT	CL/*F*	0.854 × (WT/70)^0.75^	35.9^a^	34.8%
						V_c_/*F*	10.3 × (WT/70) × (AGE/8.5) ^−0.267^	19.6^a^	
						V_p_/*F*	4.08 × (WT/70)	101.5^a^	
						Q/*F*	5.34 × (WT/70)^0.75^	—	
						K_a_	2.0 for capsule, 1.2 for sprinkle, 4.1 for tablet (fixed)	—	
						T_lag_	1.0 for sprinkle, 2.0 for tablet (fixed)	—	
Ogusu et al. (2014)	Japan (single)	237 (137/100)	827	EMIT	1CMT	CL/*F*	0.559 × (DD/1000)^0.596^ × (0.917, if female) × (1.19, if concomitant CBZ) × (1.12, if concomitant PB) × (1.43, if concomitant PHT) × (0.906, if concomitant CLB)	24.2	24.8%
						V/*F*	21.4 × (DD/1000)^1.52^	0.043	
						K_a_	0.109	0.088	
						T_lag_	3.00	—	
Ding et al. (2015)	China (multiple)	902 (547/355)	1107	FPIA	1CMT	CL/*F*	$0.3\times \left(1.43,\text{if concomitant CBZ}\right)\times {\left(\frac{\text{WT}}{70}\right)}^{0.791-\frac{0.096\times {\text{AGE}}^{8.63}}{0.802^{8.63}+{\text{AGE}}^{8.63}}}$	19.5	13.3 mg/L
							$\times \left(1+\frac{2.8\times {\text{DDW}}^{1.68}}{37.4^{1.68}+{\text{DDW}}^{1.68}}\right)$		
						V/*F*	22.2 × (WT/70)	/	
						K_a_	2.64, 1.57, 0.46 for syrup, conventional tablet and SR tablet, respectively (fixed)	/	
Rodrigues et al. (2018)	France (multiple)	98 (50/48)	325	FPIA	1CMT	CL/*F*	0.624 × (WT/70)^0.75^	33.9	15.4%
						V/*F*	13.0 × (WT/70)	/	
						K_a_	0.274	/	
Gu et al. (2021)	China (single)	313 (209/104)	375	GC	1CMT	CL/*F*	$10.4\times \left(1.25,\text{if concomitant LGT}\right)\times {\left(\frac{\text{WT}}{70}\right)}^{0.75}$ $\times \left(\frac{{\text{PMA}}^{4.17}}{33.7^{4.17}+{\text{PMA}}^{4.17}}\right)$	43.0	2.5%
						V/*F*	1680.1 × (WT/70)	92.8	
						K_a_	2.64, 1.57, 0.46 for syrup, conventional tablet and SR tablet, respectively (fixed)	/	
Teixeira-da-Silva et al. (2022)	Spain (single)	836 (451/385)	1751	FPIA	1CMT	CL/*F*	0.646 × (WT/70)^0.75^ × (AGE/15.0)^−0.0154^ × (1.640, if concomitant PHT) × (1.386, if concomitant PB) ×(1.521, if concomitant CBZ)	26.8	57.7%
						V/*F*	14.0 × (WT/70)	—	
						K_a_	2.64, 0.78, 0.38 for syrup, EC tablet and SR tablet, respectively (fixed)	—	

*BSV*, between subject variability; *CBZ*, carbamazepine; *CEDIA*, cloned enzyme donor immunoassay; *CLB*, clobazam; *CL/F*, apparent clearance (L h^−1^); *CMT*, compartment; *CO*, co-medication; *DD*, daily dose in mg day^−1^; *DDW*, daily dose in mg kg^−1^.day^−1^; *EC*, enteric coated; *EMIT*, enzyme multiplied immunoassay technique; *FPIA*, fluorescence polarization immunoassay; *GC*, gas chromatography; *k*_0_, zero order rate constant (h^−1^); *K*_*a*_, absorption rate constant (h^−1^); *LGT*, lamotrigine; *PB*, phenobarbital; *PHT*, phenytoin; *PK*, pharmacokinetics; *PMA*, post-menstrual age; *Q*, intercompartmental clearance (L h^−1^); *SR*, sustained release; *T*_*lag*_, lag time (h); *V*_*c*_ */F*, apparent volume of distribution of central compartment (L); *V/F*, apparent volume of distribution (L); *VPA*, valproic acid; *V*_*p*_ */F*, apparent volume of distribution of peripheral compartment (L); *WT*, weight.

^a^ Correlations are CL/*F* ∼ V_c_: 0.0397; CL/*F* ∼ V_p_: 0.0777; V_c_ ∼ V_p_: 0.144.

Most studies described VPA PK using a one-compartment (1CMT) model with first-order absorption (Serrano et al., 1999; Desoky et al., 2004; Jiang et al., 2007; Correa et al., 2008; Ogusu et al., 2014; Ding et al., 2015; Rodrigues et al., 2018; Gu et al., 2021; Teixeira-da-Silva et al., 2022), while only one study reported a two-compartment (2CMT) model (Williams et al., 2012). Three models were established with data from both pediatric and adult patients (Desoky et al., 2004; Ogusu et al., 2014; Teixeira-da-Silva et al., 2022). Six studies considered the effects of the formulation on the absorption rate (Desoky et al., 2004; Jiang et al., 2007; Williams et al., 2012; Ding et al., 2015; Gu et al., 2021; Teixeira-da-Silva et al., 2022), of which most used a fixed absorption rate constant (k_a_) (Desoky et al., 2004; Williams et al., 2012; Ding et al., 2015; Gu et al., 2021; Teixeira-da-Silva et al., 2022). The four covariates that most frequently influenced VPA CL/*F* were concomitant medication intake, BW, daily dose, and age, accounting for 80.0%, 70.0%, 50.0%, and 50.0% of the studies, respectively. The most commonly reported co-medications were carbamazepine, phenobarbital, phenytoin, and lamotrigine.

### 3.2 External predictive ability evaluation

#### 3.2.1 Participants

A total of 202 volunteers who met the inclusion and exclusion criteria were included, providing 255 VPA concentrations for analysis. Table 2 shows the demographic and clinical characteristics of all participants. Age, BW, VPA daily dose, and serum concentrations were 4.92 (0.17–15.00) years, 19.00 (4.00–70.00) kg, 23.40 (8.70–49.20) mg/kg/day, and 50.40 (22.60–118.50) µg/mL, respectively. A sustained-release formulation or a syrup was administered orally one, two, or three times per day. Trough concentrations were calculated under steady-state conditions. The three most commonly prescribed co-medications in our cohort were levetiracetam, oxcarbazepine, and topiramate.

**TABLE 2:** Characteristics of external evaluation dataset.

Characteristics	Number or mean ± SD	Median (range)
No. of patients (Male/Female)^a^	202 (139/63)	—
No. of samples^b^	255	—
Age (years)	5.74 ± 3.67	4.92 (0.17-15.00)
Weight (kg)	22.46 ± 12.23	19.00 (4.00-70.00)
Height (cm)	111.83 ± 25.31	108.00 (54.00-180.00)
Body surface area (m^2^)	0.83 ± 0.31	0.76 (0.24-1.85)
Formulation (syrup/sustained release tablet)^a^	194/61	—
Daily dose (mg day^−1^)	513.04 ± 234.45	480.00 (60.00-1250.00)
Daily dose (mg kg^−1^ day^−1^)	24.50 ± 7.80	23.44 (8.70-57.69)
VPA serum concentration (μg mL^−1^)	54.34 ± 17.89	50.40 (22.60-118.50)
Total Bilirubin (μmol L^−1^)	7.03 ± 2.53	6.70 (2.30-17.80)
Albumin (g L^−1^)	42.21 ± 3.65	42.10 (29.70-70.50)
Alanine aminotransferase (U L^−1^)	12.24 ± 7.12	10.00 (1.00-38.00)
Aspartate transferase (U L^−1^)	24.46 ± 7.34	24.00 (9.00-55.00)
Serum Creatinine (μmol L^−1^)	31.34 ± 8.27	29.90 (13.90-57.80)
Blood Urea Nitrogen (mmol L^−1^)	4.35 ± 1.24	4.20 (1.40-8.70)
Concomitant medication^a^	77	—
levetiracetam	27	—
oxcarbazepine	24	—
topiramate	19	—
clonazepam	12	—
lamotrigine	10	—
carbamazepine	3	—

^a^ Data are expressed as the number of patients.

^b^ Data are expressed as the number of samples.

#### 3.2.2 Prediction-based diagnostics

**FIGURE 2:**
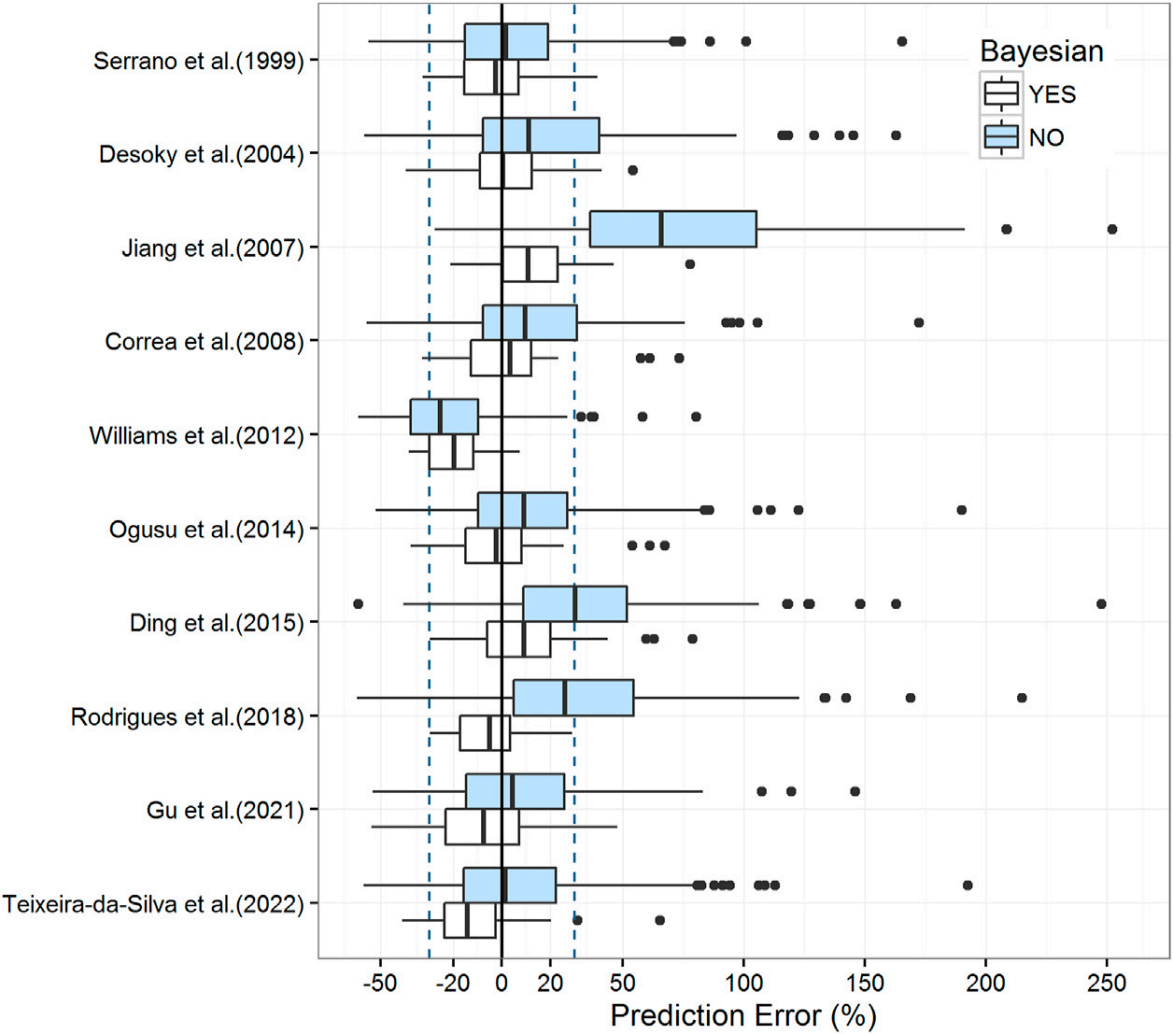
Box plots of the prediction error with or without Bayesian forecasting for published population pharmacokinetic models. The blue box represents predictions without *prior* information, while the white box represents predictions with one *prior* observation. Black solid lines and blue dotted lines are reference lines indicating PE% of 0% and ±30%, respectively.

**FIGURE 3:**
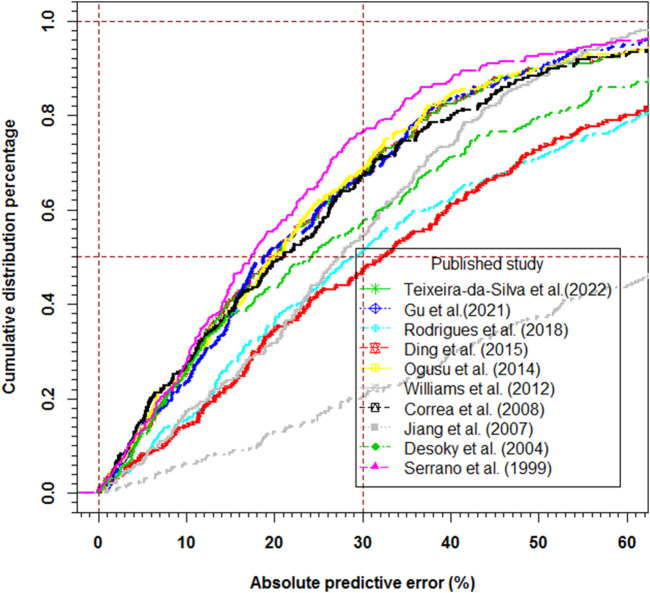
The cumulative distribution percentage plots of absolute value of prediction error.

The prediction-based diagnostic results are listed in Table 3. The models proposed by Serrano et al. (1999), Desoky et al. (2004), Correa et al. (2008), Ogusu et al. (2014), Gu et al. (2021), and Teixeira-da-Silva et al. (2022) met the criteria mentioned above (MDPE ≤ ± 15%, MAPE ≤30%, F_20_ > 35%, and F_30_ > 50%). The model proposed by Serrano et al. (1999) (MDPE, 1.40%; MAPE, 17.38%; F_20_, 55.69%; F_30_, 76.47%) performed the best. The predictive abilities of these models are displayed in box plots (Figure 2) and cumulative distribution curve plots (Figure 3).

**TABLE 3:** Results of the prediction-based metrics with or without *prior* observation.

Models	Without *prior* observation				With one *prior* observation			
	MDPE	MAPE	F_20_	F_30_	MDIPE	MAIPE	IF_20_	IF_30_
Serrano et al. (1999)	1.40	17.38	55.69	76.47	−2.62	9.46	71.70	90.57
Desoky et al. (2004)	11.25	24.28	43.53	57.25	0.47	10.64	69.81	83.02
Jiang et al. (2007)	66.84	66.84	12.94	20.39	10.98	12.07	66.04	81.13
Correa et al. (2008)	9.37	21.03	49.02	67.06	3.32	12.06	73.58	86.79
Williams et al. (2012)	−25.92	27.70	31.76	54.51	−19.61	19.61	52.83	77.36
Ogusu et al. (2014)	8.84	19.90	50.20	68.63	−2.23	11.11	71.70	84.91
Ding et al. (2015)	30.24	32.14	34.51	47.06	9.24	17.14	64.15	88.68
Rodrigues et al. (2018)	26.13	29.36	36.08	50.98	−5.02	13.39	71.70	100
Gu et al. (2021)	4.35	18.74	51.76	67.06	−7.61	14.56	62.26	79.25
Teixeira-da-Silva et al. (2022)	1.34	19.80	50.20	67.06	−14.20	15.83	56.60	81.13
Impact of protein binding modeling strategy								
One-binding site model	3.48	19.38	51.37	68.24	−3.05	16.29	61.70	85.11
Langmuir equation	0.82	20.49	47.84	67.45	−1.73	4.06	97.87	100
Dose-dependent maximum effect model	−1.10	27.67	39.61	52.94	−4.43	5.47	97.87	97.87
Linear non-saturable binding equation	−0.19	19.8	50.20	72.16	−2.51	5.51	97.87	100
The simple exponent model	1.50	17.68	56.47	72.94	−2.79	6.47	97.87	97.87

*F*_*20*_, *F*_*30*_, the percentages of prediction error within 20% and 30%, respectively; *MAPE*, median absolute prediction error; *MDPE*, median prediction error; *IF*_*20*_, *IF*_*30*_, the percentages of individual prediction error within 20% and 30%, respectively; *MAIPE*, median absolute individual prediction error; *MDIPE*, median individual prediction error.

#### 3.2.3 Simulation-based diagnostics

The results of pcVPC indicated a significant deviation between observations and simulations among the most involved models (Supplementary Figure S1). A clear pattern of over- or under-prediction of true variability among subjects was observed, except in the relatively superior model of prediction-based diagnostics developed by Serrano et al. (1999). However, the NPDE results of the model proposed by Serrano et al. (1999) were not as accurate as those of pcVPC, which failed to obey a normal distribution, especially for the description of variance. For the global test (Supplementary Table S2), all models were statistically rejected (*p*-values >0.05). The NPDE results are shown in Supplementary Figure S2 and Supplementary Table S2.

#### 3.2.4 Bayesian forecasting

In Bayesian forecasting, the prediction accuracy was substantially enhanced by one *prior* observation in most models (Figure 2; Table 3), which highlighted the usefulness of popPK modeling combined with Bayesian estimation in VPA dosage adjustments. Due to the limitation of the sample size, the Bayesian forecasting results of two or three *prior* observations were not available in this study. As model predictability reaches a stable state after two or three *prior* observations (Zhao et al., 2016a; Mao et al., 2018), this limitation might have caused fluctuation in model accuracy, such as those observed for models proposed by Gu et al. (2021) and Teixeira-da-Silva et al. (2022).

### 3.3 The impact of protein binding modeling strategy

**FIGURE 4:**
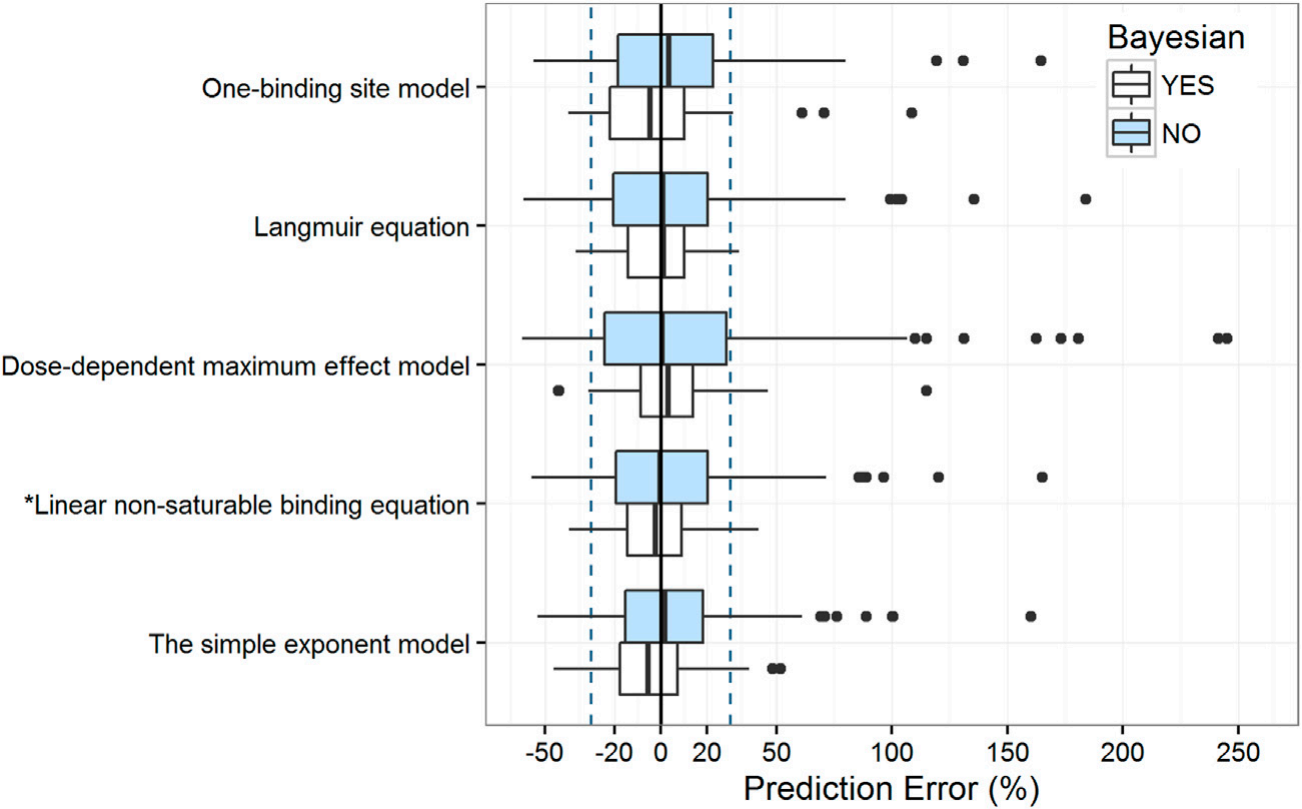
Box plots of the prediction error with or without Bayesian forecasting for five protein binding models. The blue box represents predictions without *prior* information, while the white box represents predictions with one *prior* observation. Black solid lines and blue dotted lines are reference lines indicating PE% of 0% and ±30%, respectively. The model with an asterisk (*) performed the best.

The estimated parameters of the five protein-binding modeling strategies based on our evaluation dataset are shown in Supplementary Table S3. The objective function value decreased dramatically as non-linearity was involved in modeling, except in the dose-dependent maximum effect model. Moreover, the aforementioned assessments were employed to evaluate the predictive ability of these models. Results for these metrics are presented in Table 3 and Figure 4.

The results of the prediction-based diagnostics showed that the linear non-saturable binding equation and the simple exponent model performed better than the other models. After Bayesian forecasting, the prediction accuracy improved substantially with one *prior* observation. The simulation-based diagnostics (Supplementary Table S2, Supplementary Figures S3, S4) indicated that more covariates should be identified to quantify the variabilities.

Although the simple exponent model had a satisfactory predictive ability, the equation did not describe the non-linear properties of the VPA PK process (Supplementary Figure S5). According to the tendency plot results, the dose-dependent maximum effect model describes the non-linear property of the VPA PK process. However, regarding the empirical-estimated parameters and center-related variability, the dose-dependent maximum effect model did not had a satisfactory simulation-based predictive ability. Therefore, the linear non-saturable binding equation is more suitable for modeling the non-linear kinetics of VPA according to the saturation of protein-binding.

## 4 Discussion

Although several studies have reported popPK characteristics in children with epilepsy, the predictive ability of these models have not been fully assessed. In this study, we systematically evaluated 10 published popPK models in children with epilepsy. Although six models met the criteria (MDPE ≤ ± 15%, MAPE ≤30%, F_20_ > 35%, and F_30_ > 50%) in prediction-based diagnostics, large variabilities existed in simulation-based diagnostics, indicating the discrepancies across centers, especially for variance. With *prior* observations available, the performance of popPK models significantly improves, indicating that Bayesian forecasting substantially improves the prediction accuracy of the popPK model (Zhao et al., 2016a; Mao et al., 2018; Cai et al., 2020).

Given the similarity of participants’ race, prescription regimen, dietary habits, and genetics, models established in populations with similar evaluation datasets might have superior predictive ability. However, models developed for East Asians (Jiang et al., 2007; Ding et al., 2015) did not show any advantages in the evaluation. The typical CL/*F* values in those studies were 0.18 and 0.24 L/h, respectively, which are lower than that of our base model (0.31 L/h), resulting in an overestimation of concentration. Moreover, the best method for prediction-based diagnostics and simulation-based pcVPC was performed in Spain, with 255 patients and 770 samples (Serrano et al., 1999). Indeed, no obvious relationship between superior predictive performance and sample size or ethnicity was observed in our study.

VPA is highly bind to plasma proteins, and the binding sites can be saturated as total VPA concentration increases, thus following non-linear PK. To capture this phenomenon, five candidate studies included dose as a covariate in the models to characterize the non-linear relationship between VPA dose and CL/*F* (Serrano et al., 1999; Desoky et al., 2004; Correa et al., 2008; Ogusu et al., 2014; Ding et al., 2015). Most of them chose a simple exponent model (Serrano et al., 1999; Desoky et al., 2004; Correa et al., 2008; Ogusu et al., 2014), while only a study by Ding et al. (2015) proposed a dose-dependent maximum effect (DDE) model.

Moreover, the incorporation of the VPA daily dose is controversial with regard to its influence on CL/*F*. In patients with a higher clearance rate, lower drug concentrations may be present. Therefore, they require higher TDM-guided doses. Regarding the complicated TDM effect, the simple exponential model may not be suitable for describing non-linear PK profiles (Ahn et al., 2005). In addition, the simple exponent model was insufficient in describing the non-linear correlation between the daily dose and CL/*F* as indicated by the tendency plot results. Therefore, although model predictive ability may be improved when considering the effect of the daily dose, it is not a suitable strategy for modeling PK non-linearity by adding daily doses to CL/*F* (Vucicevic et al., 2009; Teixeira-da-Silva et al., 2022). In fact, as the VPA daily dose was the goal of prediction, it should not be used as a covariate for prediction.

Regardless of whether the DDE model or the simple exponent model were data-driven empirical models, the transferability of these models may be influenced by center-related factors, which might be partly due to the differences in study design (such as age and dosage regimen) and retained covariates between different clinical sites. Adding covariates based on the mechanisms of PK processes may assist in improvement of a model’s predictive ability (Danhof et al., 2008). In addition, only free VPA can access the central nervous system, where its pharmacological action occurs. Therefore, the estimation of the free VPA concentration based on the understanding of serum protein binding is essential (Kodama et al., 1992b). However, as a low extraction ratio drug, the effective concentration of VPA does not only depend on protein binding, but also on the intrinsic ability of the eliminating organ. Saturation or competition would result in dose-normalized reduction of the total concentration whereas the free concentration may remain unchanged (Benet and Hoener, 2002).

The one-binding site model (Dutta et al., 2007), Langmuir equation (Ueshima et al., 2008), and linear non-saturable binding equation (Gu et al., 2021) have been built based on the understanding of the non-linear relationship between the total and free serum concentrations of VPA. Age, ultrafiltration temperature, albumin level, and the existence of various metabolites affect VPA binding in a population of patients (Kodama et al., 1992b). Age is positively correlated with the VPA free fraction (Zaccara et al., 1988). Therefore, a one-binding site model built on adult patients may not be suitable for the pediatric population. The linear non-saturable binding equation incorporating the slope of non-saturable protein binding may be superior to the Langmuir equation, which has been confirmed in a previous study (Gu et al., 2021).

BW is a body size indicator associated with the functionality of the liver, which is responsible for VPA metabolism. It is one of the most common covariates considered in final models, accounting for nearly 62.5% of candidate studies (Xu et al., 2018). Although the 3/4 allometric exponent model has been universally applied to scale clearance (Anderson et al., 2007; Holford et al., 2013), the value of 0.75 in this approach might not be reliable for estimating clearance in pediatric patients (Mahmood, 2006; Peeters et al., 2010). Moreover, age is an important maturation marker, and some studies have shown that VPA CL/*F* varies with age in children (Chiba et al., 1985; Cloyd et al., 1993). However, age was not included in half of the selected models, including the superior model proposed by Serrano et al. (1999).

Both age and BW reportedly determine maturation in children <2 years old, whereas BW is the most important factor influencing CL/*F* in children ≥2 years old (Ding et al., 2015; Gu et al., 2021). The age-dependence of PK in young children may be partially due to the maturation of UGT enzymes that mediate VPA elimination. The activities of UGT1A9 and UGT1A6 reach adult levels at 2 years and 14 months of age, respectively (Choonara et al., 1989; Miyagi and Collier, 2011). The ability of another UGT enzyme involved in VPA elimination, UGT2B7, reaches adult levels between two and 6 months of age (Ebner and Burchell, 1993). The median age in each of the studies analyzed here was above 2 years. That explains why BW was the most common covariate in the final models.

In addition, several other center-based factors could have resulted in inter-study variability and affected the model’s predictive performance. The VPA bioassay is one of the most frequently mentioned factors. Three immunoassay methods were used in the candidate publications, including FPIA, EMIT, and CEDIA, whereas a GC technique was used for our dataset. The analytical performance of these immunoassays was reported to be practically equivalent to that of chromatographic methods (Johannessen and Johannessen, 2003). Correlation coefficients for ultraperformance liquid chromatography mass spectrometry *versus* FPIA, and high-performance liquid chromatography has been reported as 0.989, and 0.987, respectively; Bland-Altman analysis has also shown these methods to be comparable (Zhao et al., 2016b), indicating their agreement. Although EMIT overestimates VPA levels compared with chromatographic methods (Xia et al., 2021), no clear correlation between model predictive ability and bioassay method was observed (Zang et al., 2022).

Pharmacogenetics may also contribute to drug PK variability. VPA metabolism is related to several metabolic enzymes and transporters, including UGTs, CYPs, ATP-binding cassette (ABC) transporters, and monocarboxylate transporters (MCTs). Genetic polymorphisms of these enzymes and transporters may influence the PKs and VPA concentrations (Hung et al., 2011; Mei et al., 2018). However, few researchers have regarded polymorphisms as covariates of the popPK characteristics of VPA (Jiang et al., 2009; Mei et al., 2018; Xu et al., 2018), and the findings remain controversial in pediatric patients. In our previous study, 11 single-nucleotide polymorphisms in UGT2B7, UGT1A6, and CYP2C9 were investigated, revealing no significant influence of any of them on VPA responsiveness (Liu et al., 2020). As pharmacogenomic considerations have not been verified in clinical practice, genotyping is not routinely performed in VPA therapy, and genetic polymorphisms were not available in a part of our external evaluation dataset. The role of pharmacogenetics in model predictive ability was not explored in this work, and needs further clarification in future research, specifically in children.

This study has several limitations. First, the external dataset consisted of a fraction of participants (202 children) from a single center, which could limit statistical power. In addition, the collected data were mostly at trough concentrations; therefore, parameters for the absorption and distribution stages could not be obtained precisely. Furthermore, among the candidate studies, five reported traditional ASMs, such as phenobarbital, phenytoin, and carbamazepine, have enzymatic induction effects that can enhance VPA metabolism in children. However, novel ASMs (levetiracetam, oxcarbazepine, and topiramate) had been commonly prescribed for polytherapy in our population. Owing to the low proportion of patients treated concomitantly with classical enzyme inducers, drug-drug interactions were applied as a predictive factor, which may have led to misspecification to some extent. Furthermore, the inconsistency of bioassays may result in systematic biases.

## 5 Conclusion

The predictive performance of most selected popPK models of VPA in children with epilepsy was unsatisfactory and diverse, and the direct extrapolation of these models to the clinical application should be performed with caution. Describing the non-linear kinetics of VPA based on the mechanisms of PK processes may enhance model predictive ability. Importantly, the linear non-saturable binding equation is more suitable for modeling the non-linearity in terms of protein-binding saturation. Moreover, Bayesian forecasting with *prior* observations led to an improvement in model fitness.

## Principal investigator statement

The authors confirm that the Principal Investigator for this paper is Maochang Liu and that he had direct clinical responsibility for patients.

## Data Availability

The raw data supporting the conclusion of this article will be made available by the authors, without undue reservation.
