# Supplementary material for: Modeling the protein binding non-linearity in population pharmacokinetic model of valproic acid in children with epilepsy: a systematic evaluation study
Source: Front Pharmacol. 2023 Oct 6;14:1228641. doi: 10.3389/fphar.2023.1228641 (PMC10587682; doi:10.3389/fphar.2023.1228641)
Supplement: Supplementary file 1 [file DataSheet4.docx]

# Electronic Supplementary Material

## Supplementary Figure. S1A

| 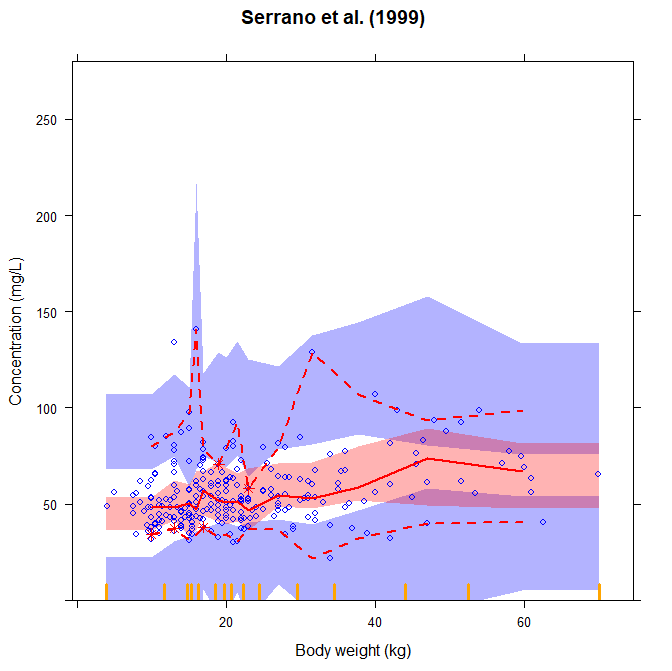 | **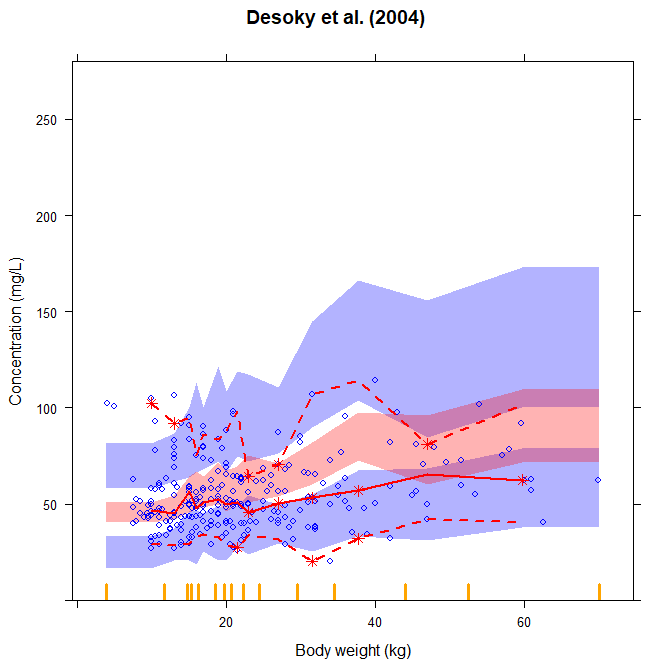** |
| --- | --- |

## Supplementary Figure. S1B

| **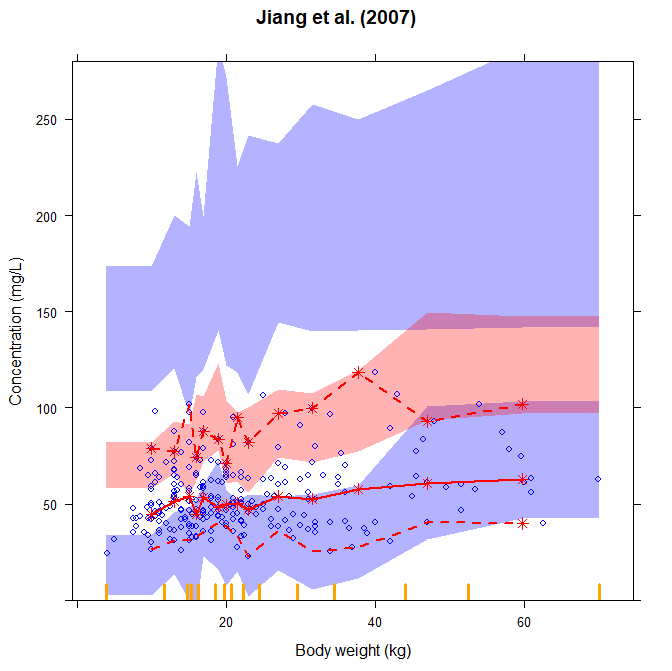** | **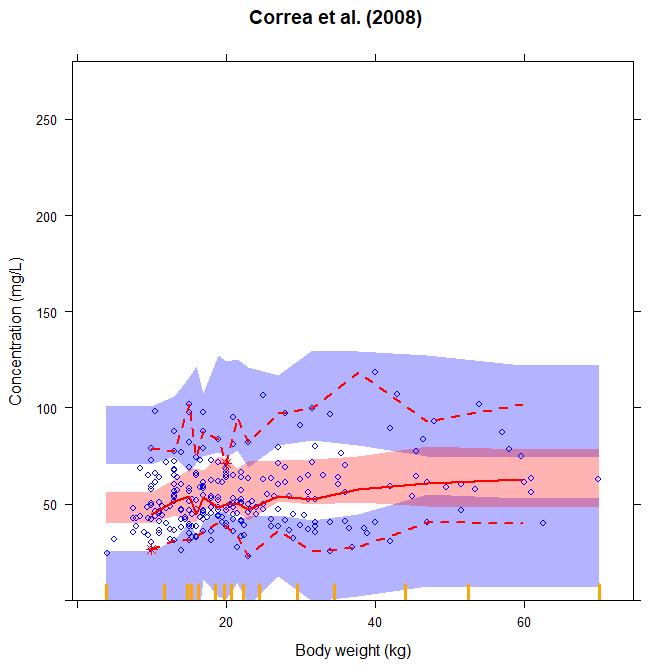** |
| --- | --- |

## Supplementary Figure. S1C

| 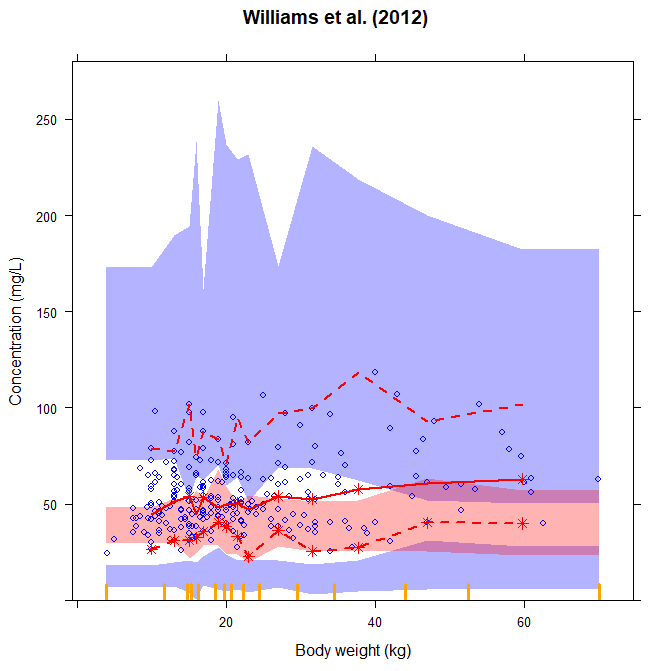 | **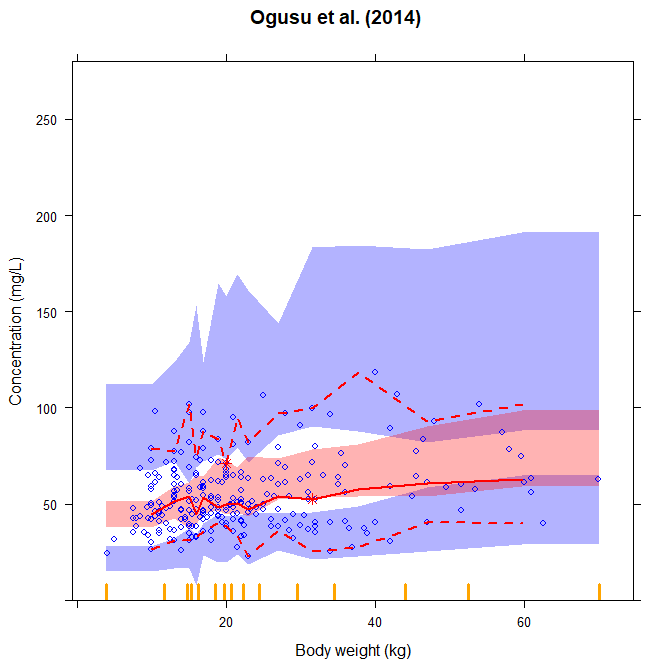** |
| --- | --- |

## Supplementary Figure. S1D

| 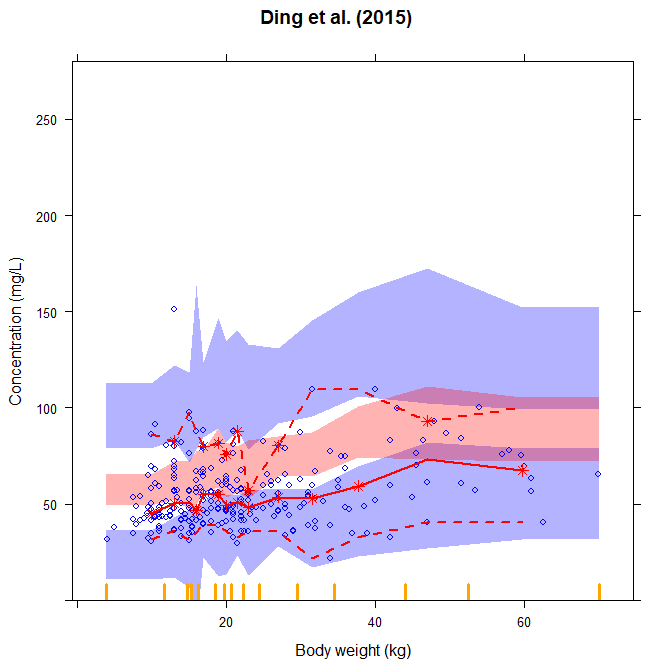 | **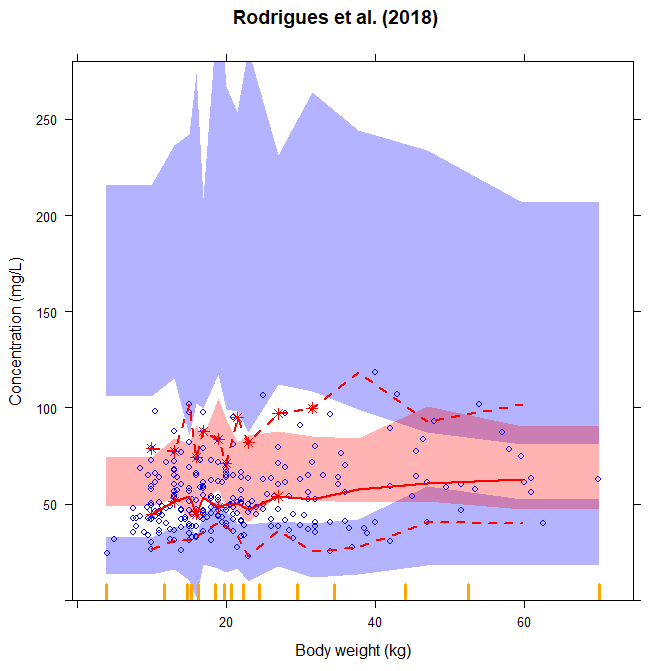** |
| --- | --- |

## Supplementary Figure. S1E

| 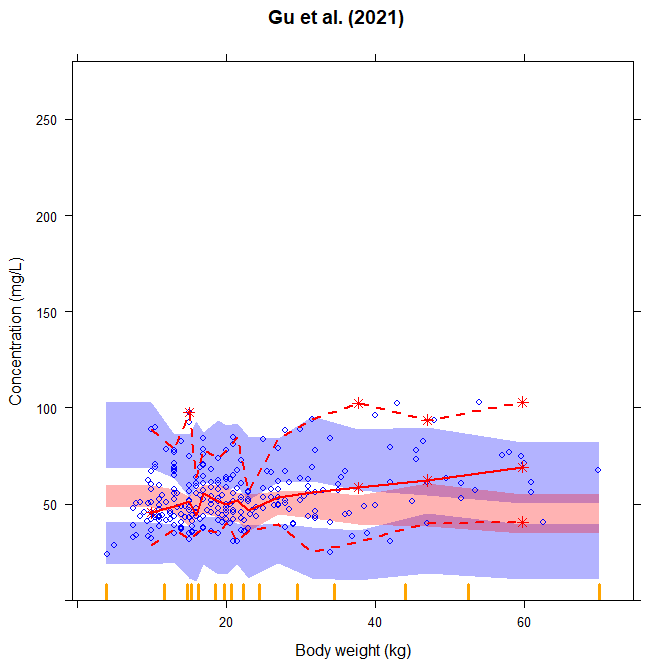 | **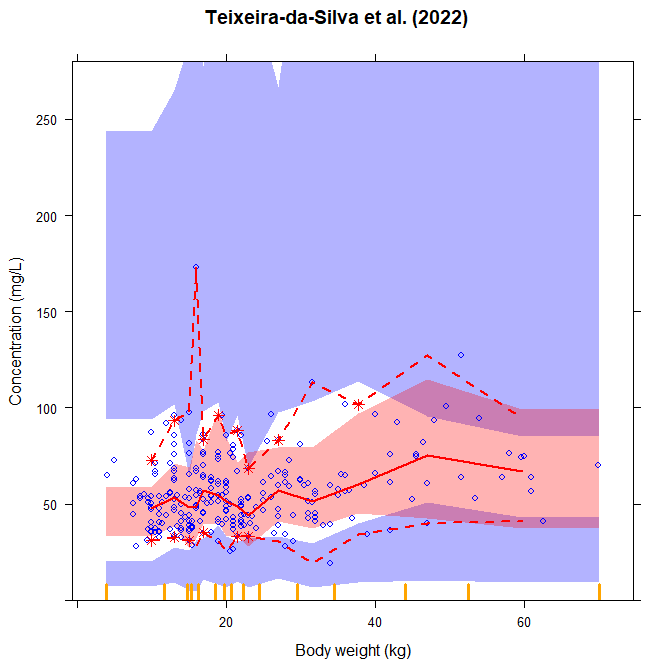** |
| --- | --- |

## Supplementary Figure. S2A


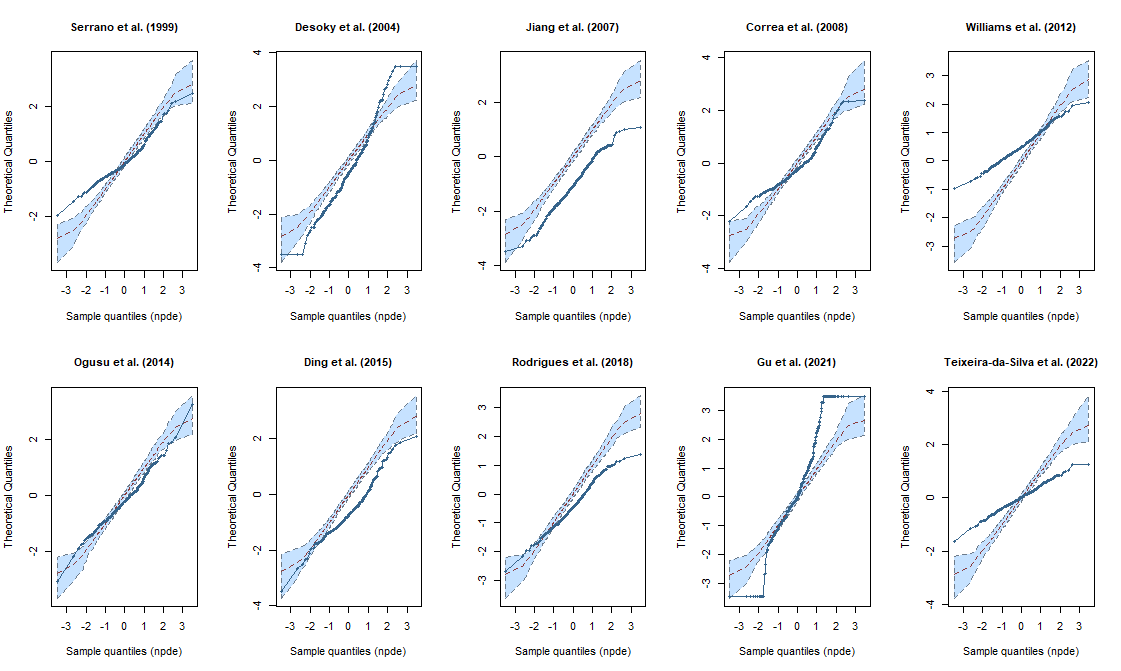


##
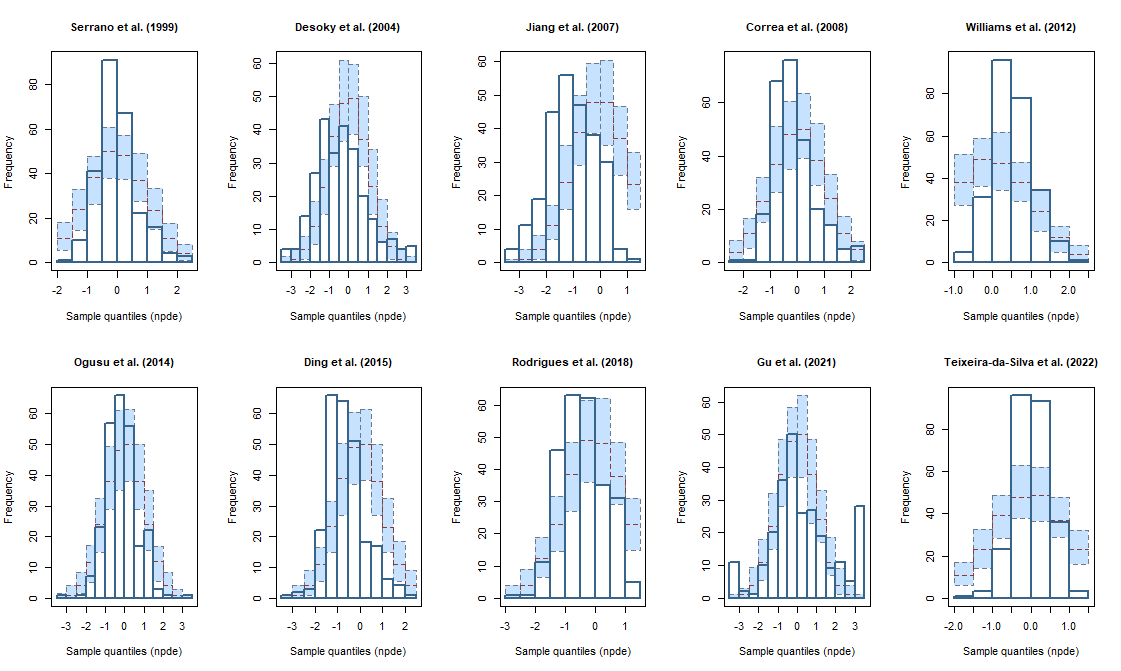
Supplementary Figure. S2B

##
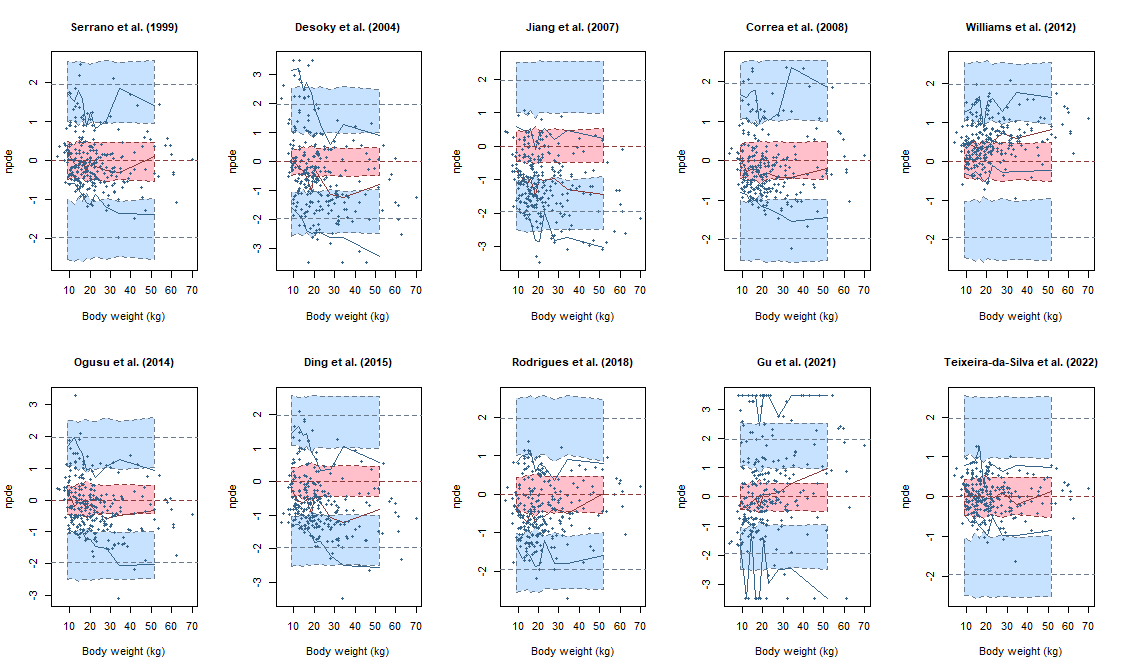
Supplementary Figure. S2C

##
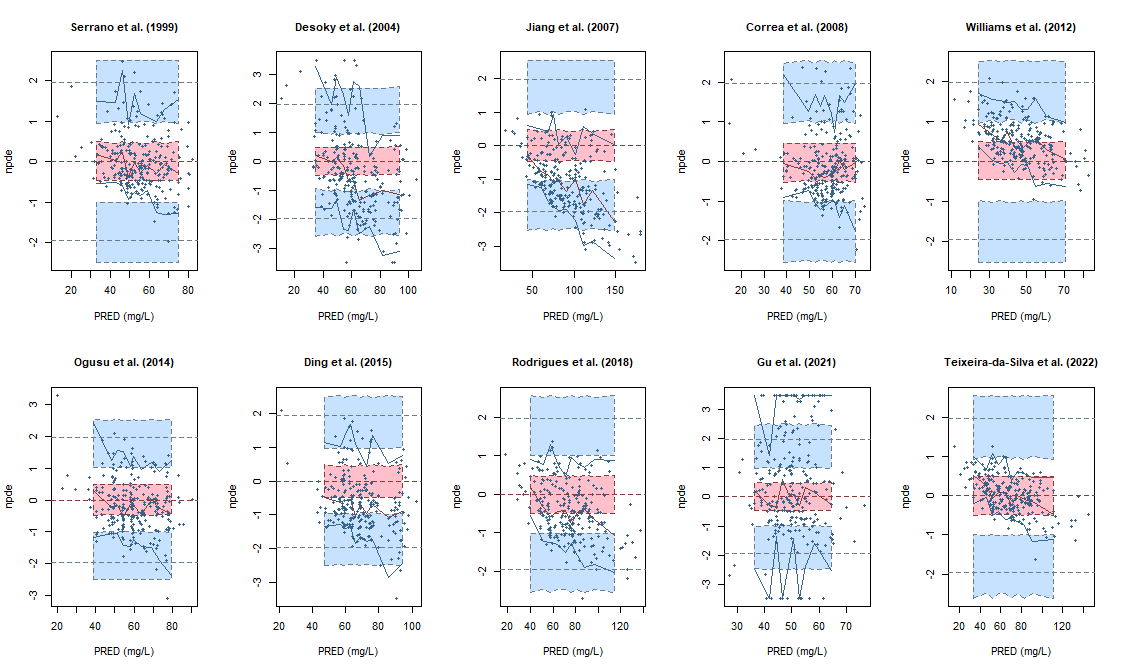
Supplementary Figure. S2D
