# Supplementary material for: Modeling the protein binding non-linearity in population pharmacokinetic model of valproic acid in children with epilepsy: a systematic evaluation study
Source: Front Pharmacol. 2023 Oct 6;14:1228641. doi: 10.3389/fphar.2023.1228641 (PMC10587682; doi:10.3389/fphar.2023.1228641)
Supplement: Supplementary file 2 [file DataSheet3.docx]

**Electronic Supplementary Material**

**Supplementary Table S1 Demographic characteristics for published population pharmacokinetic models**

| Study  (publication year) | Country | No. of patients  (male/female) | Age  median (range) / mean ± SD (years) | Body weight  median (range) / mean ± SD (kg) | Dose range  (mg/kg/d) | Formulation | Total samples  [per patients] | VPA assay  [LOQ] |
| --- | --- | --- | --- | --- | --- | --- | --- | --- |
| Serrano *et al.* (1999)[1] | Spain | 255 (128/127) | 7.8 [0.1-14.0] | 31.3 [4.0-74.0] | 24.2 [15.7-50.0] mg/(kg·d) | syrup, EC tablet | 770 [2-5] | FPIA (CV < 10%) |
| Desoky *et al.* (2004)[2] | Egyptian | 81 (52/29) | 20 [3-58] | 48.4 [15-75] | 18.8 [1.4-44.0] mg/(kg·d) | EC tablets,  oral solution | 81[1] | FPIA (CV < 10%) |
| Jiang *et al.* (2007)[3] | China | 317 (195/122) | 8.8 [0.3-16.0] | 33.7 [6.0-113.0] | 21.8 [3.0-80.8] mg/(kg·d) | NR (have SR  tablet) | 624 [1-10] | FPIA (CV < 4%) |
| Correa *et al.* (2008)[4] | Mexico | 110 (63/47) | 7.0 ± 4.5 [0.5-17.0] | 27.1 ± 16.5 [8.0-68.5] | 29.3 [9.1-70.0] mg/(kg·d) | syrup, tablet | 119 | CEDIA (CV < 10%) |
| Williams *et al.* (2012)[5] | USA | 52 (36/16) | 8.5 [1.0-17.0] | NR | 23.0 [3.0-60.0] mg/(kg·d) | syrup, capsule, EC tablet, EC  Sprinkle, IV | 231 [1-15] | FPIA (CV < 5%)  VITROs VALP assay (CV < 5%) |
| Ogusu *et al.* (2014)[6] | Japan | PK analysis: 237 (137/100)  PK-PD  analysis: 169 (102/67) | 17.2 ± 8.3  [2.2-52.2]  18.0 ± 7.8  [3.0-52.2] | 48.8 ± 20.9  [9.6-120.5]  51.0 ± 20.1  [13.0–120.5] | 934.3 ± 540.2 [100-2600] mg/d  903.8 ± 502.7 [100-2600] mg/d | SR tablet | 827  42 γ-GT levels | EMIT  (CV <10%)  [1 mg/L] |
| Ding *et al.* (2015) [7] | China | 902 (547/355) | 5.7 ± 3.8 [0.3-14.0] | 21.6 ± 11.9 [2.6-70.0] | 27.1 [5.1-63.2] mg/(kg·d) | syrup, tablet, SR tablet | 1107 | FPIA (CV < 10%) |
| Rodrigues *et al.* (2018)[8] | France | 98 (50/48) | 6.0 [1.0-17.6] | 20.0 [7.0-69.0] | 21.9 [3.2-40.0] mg/(kg·d) | SR granule | 325 | FPIA (CV < 5%) |
| Gu *et al.* (2021)[9] | China | 313 (209/104) | 5.96 [0.31-15.89] | 21.20 [7.00-94.50] | 412.66 [80.00-1250.00] mg/d | syrup, tablet, SR tablet | 375 | GC [3.23 mg/L] |
| Teixeira-da-Silva *et al.* (2022)[10] | Spain | 836 (451/385) | 32.4 [0.1-89.4] | ﻿60.0 [6.7-125.0] | 1107.1 ± 587.0 [150.0-4500.0] mg/d | syrup, EC tablet, SR tablet | 1751 | FPIA (CV < 10%) |

**Supplementary Table S2 Statistic test results of normalized prediction distribution errors (NPDE) diagnostics**

| **Models** | **Mean (SE)** | **Variance (SE)** | **Kurtosis** | **Skewness** | **Wilcoxon signed rank test^†^** | **Fisher test^†^** | **Shapiro-Wilks test^†^** | **Global test^†^** |
| --- | --- | --- | --- | --- | --- | --- | --- | --- |
| *Published Studies* |  |  |  |  |  |  |  |  |
| Serrano *et al*. (1999) | 0.0056 (0.04) | 0.45 (0.04) | 1.12 | 0.77 | 0.89 | 0.00 | 0.00 | 0.00 |
| Desoky *et al*. (2004) | -0.39 (0.08) | 1.76 (0.16) | 0.45 | 0.55 | 0.00 | 0.00 | 0.00 | 0.00 |
| Jiang *et al*. (2007) | -1.05 (0.06) | 0.79 (0.07) | -0.30 | -0.08 | 0.00 | 0.01 | 0.41 | 0.00 |
| Correa *et al*. (2008) | -0.14 (0.05) | 0.60 (0.05) | 1.12 | 0.95 | 0.005 | 0.00 | 0.00 | 0.00 |
| Williams *et al*. (2012) | 0.52 (0.03) | 0.27 (0.02) | 0.09 | 0.22 | 0.00 | 0.00 | 0.57 | 0.00 |
| Ogusu *et al*. (2014) | -0.17 (0.05) | 0.66 (0.06) | 1.31 | 0.40 | 0.00 | 0.00 | 0.002 | 0.00 |
| Ding *et al.* (2015) | -0.64 (0.05) | 0.71 (0.06) | 0.91 | 0.54 | 0.00 | 0.00 | 0.00 | 0.00 |
| Rodrigues *et al.* (2018) | -0.42 (0.05) | 0.54 (0.05) | -0.36 | 0.09 | 0.00 | 0.00 | 0.16 | 0.00 |
| Gu *et al.* (2021) | 0.29 (0.11) | 2.84 (0.25) | -0.08 | 0.18 | 0.01 | 0.00 | 0.00 | 0.00 |
| Teixeira-da-Silva *et al.* (2022) | 0.02 (0.03) | 0.20 (0.02) | 0.36 | -0.09 | 0.54 | 0.00 | 0.75 | 0.00 |
| *Impact of protein binding modeling strategy* | | | | | | | | |
| One-binding site model | 0.19 (0.06) | 0.88 (0.08) | 0.16 | 0.22 | 0.00 | 0.19 | 0.03 | 0.00 |
| Langmuir equation | 0.28 (0.06) | 1.00 (0.09) | 0.29 | 0.28 | 0.00 | 0.94 | 0.02 | 0.00 |
| Dose-dependent maximum effect model | 0.16 (0.06) | 0.99 (0.09) | 0.03 | 0.24 | 0.01 | 0.93 | 0.10 | 0.03 |
| Linear non-saturable binding equation | 0.29 (0.06) | 0.97 (0.09) | 0.29 | 0.34 | 0.00 | 0.80 | 0.01 | 0.00 |
| The simple exponent model | 0.16 (0.06) | 0.99 (0.09) | 0.42 | 0.49 | 0.01 | 0.99 | 0.00 | 0.00 |

**^†^**Data is expressed as *P* value

**Supplementary Table S3 Parameter estimates of base model and five protein binding models**

| **Parameters** | **Base model** | **Model Ⅰ: One-binding site model** | **Model Ⅱ: Langmuir equation** | **Model Ⅲ: Dose-dependent maximum effect model** | **Model Ⅳ: Linear non-saturable binding equation** | **Model Ⅴ: The simple exponent model** |
| --- | --- | --- | --- | --- | --- | --- |
| Objective function value | 1752.1 | 1676.7 | 1647.8 | 1773.0 | 1617.5 | 1615.9 |
| CL_p_/*F* (L/h) | 0.311 (4.6) | 132.0 (7.3) | 3.4 (4.6) | 0.0815 (33.7) | 4.4 (4.0) | 0.331 (2.1) |
| V/*F* (L) | 27.8 (8.8) | 13000 (13.8) | 341.0 (5.5) | 52.3 (12.6) | 492.0 (5.8) | 17.2 (20.0) |
| CL_DD | / | / | / | / | / | 0.658 (7.7%) |
| BSV_CL/*F* (RSE%) | 45.7 (8.1) | 47.2 (13.0) | 51.9 (8.1) | 68.6 (20.5) | 55.5 (10.7) | 24.3 (11.5) |
| BSV_V/*F* (RSE%) | 68.6 (8.9) | 68.9 (6.5) | 72.2 (5.5) | 45.9 (12.4) | 77.7 (8.9) | 45.1 (15.9) |
| Residual Error | 8.9 (29.1) | 26.1 (5.1) | 12.5 (11.2) | 11.7 (17.9) | 13.9 (11.0) | 14.1 (11.9) |
|  | 6.3 mg/L (35.6) | 2.6 mg/L (107.3) | 4.3 mg/L (37.3) | 6.8 mg/L (31.9) | 4.1 mg/L (25.6) | 4.2 mg/L (35.7) |

Estimates are expressed as estimate (% standard error)

*BSV*, between subject variability; *CL/F*, apparent clearance; *DD*, daily dose; *K_a_*, absorption rate constant; *RSE*, relative standard error; *V/F*, apparent central volume of distribution.

*K_a_* was fixed to 2.64, 1.57, 0.46 for syrup, conventional tablet and SR tablet, respectively

**REFERENCE**

1 Serrano BB, Garcia Sanchez MJ, Otero MJ, Buelga DS, Serrano J, Dominguez-Gil A (1999) Valproate population pharmacokinetics in children. Journal of clinical pharmacy and therapeutics 24 (1): 73-80 DOI 10.1046/j.1365-2710.1999.00202.x

2 Desoky ESE, Fuseau E, Amry SED, Cosson V (2004) Pharmacokinetic modelling of valproic acid from routine clinical data in Egyptian epileptic patients. European journal of clinical pharmacology 59 (11): 783-790 DOI 10.1007/s00228-003-0699-7

3 Jiang D-c, Wang L, Wang Y-q, Li L, Lu W, Bai X-r (2007) Population pharmacokinetics of valproate in Chinese children with epilepsy. Acta pharmacologica Sinica 28 (10): 1677-1684 DOI 10.1111/j.1745-7254.2007.00704.x

4 Correa T, Rodriguez I, Romano S (2008) Population pharmacokinetics of valproate in Mexican children with epilepsy. Biopharmaceutics & drug disposition 29 (9): 511-520 DOI 10.1002/bdd.636

5 Williams JH, Jayaraman B, Swoboda KJ, Barrett JS (2012) Population pharmacokinetics of valproic acid in pediatric patients with epilepsy: considerations for dosing spinal muscular atrophy patients. Journal of clinical pharmacology 52 (11): 1676-1688 DOI 10.1177/0091270011428138

6 Ogusu N, Saruwatari J, Nakashima H, Noai M, Nishimura M, Deguchi M, Oniki K, Yasui-Furukori N, Kaneko S, Ishitsu T, Nakagaswa K (2014) Impact of the superoxide dismutase 2 Val16Ala polymorphism on the relationship between valproic acid exposure and elevation of gamma-glutamyltransferase in patients with epilepsy: a population pharmacokinetic-pharmacodynamic analysis. PloS one 9 (11): e111066 DOI 10.1371/journal.pone.0111066

7 Ding J, Wang Y, Lin W, Wang C, Zhao L, Li X, Zhao Z, Miao L, Jiao Z (2015) A population pharmacokinetic model of valproic acid in pediatric patients with epilepsy: a non-linear pharmacokinetic model based on protein-binding saturation. Clinical pharmacokinetics 54 (3): 305-317 DOI 10.1007/s40262-014-0212-8

8 Rodrigues C, Chhun S, Chiron C, Dulac O, Rey E, Pons G, Jullien V (2018) A population pharmacokinetic model taking into account protein binding for the sustained-release granule formulation of valproic acid in children with epilepsy. European journal of clinical pharmacology 74 (6): 793-803 DOI 10.1007/s00228-018-2444-2

9 Gu X, Zhu M, Sheng C, Yu S, Peng Q, Ma M, Hu Y, Li Z, Jiao Z, Zhou B (2021) Population pharmacokinetics of unbound valproic acid in pediatric epilepsy patients in China: a protein binding model. European journal of clinical pharmacology 77 (7): 999-1009 DOI 10.1007/s00228-020-03080-y

10 Teixeira-da-Silva P, Perez-Blanco JS, Santos-Buelga D, Otero MJ, Garcia MJ (2022) Population Pharmacokinetics of Valproic Acid in Pediatric and Adult Caucasian Patients. Pharmaceutics 14 (4) DOI 10.3390/pharmaceutics14040811
