# Supplementary material for: Modeling the protein binding non-linearity in population pharmacokinetic model of valproic acid in children with epilepsy: a systematic evaluation study
Source: Front Pharmacol. 2023 Oct 6;14:1228641. doi: 10.3389/fphar.2023.1228641 (PMC10587682; doi:10.3389/fphar.2023.1228641)
Supplement: Supplementary file 3 [file DataSheet5.docx]

# Electronic Supplementary Material

## Supplementary Figure. S3A

| 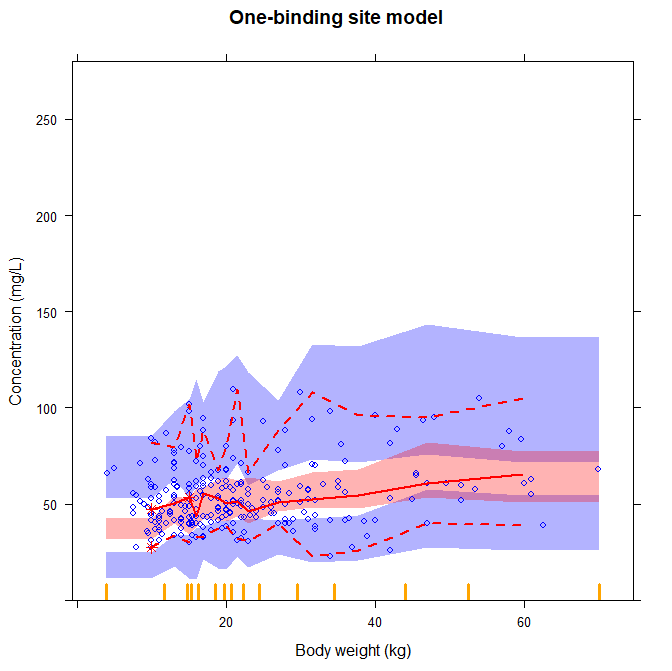 | 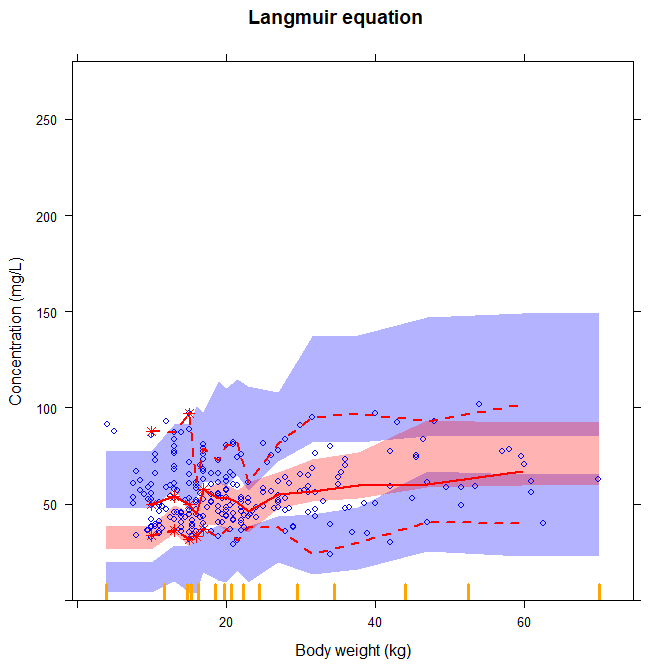 |
| --- | --- |

## Supplementary Figure. S3B

| 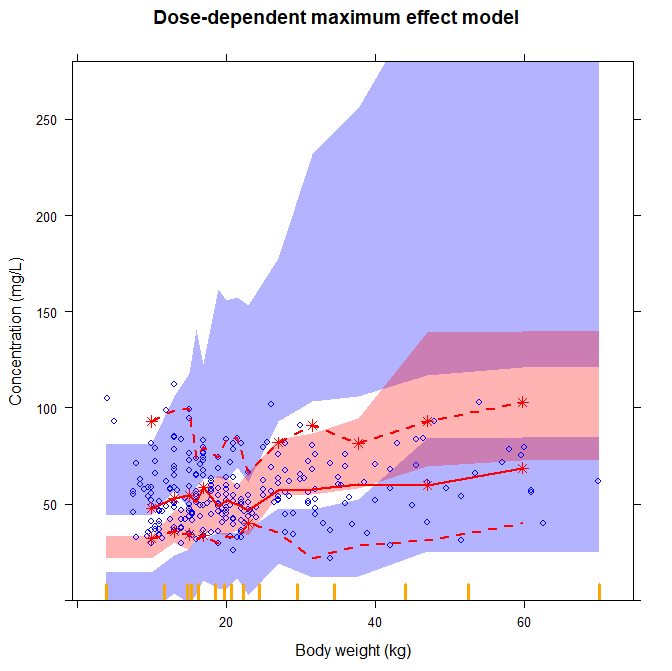 | 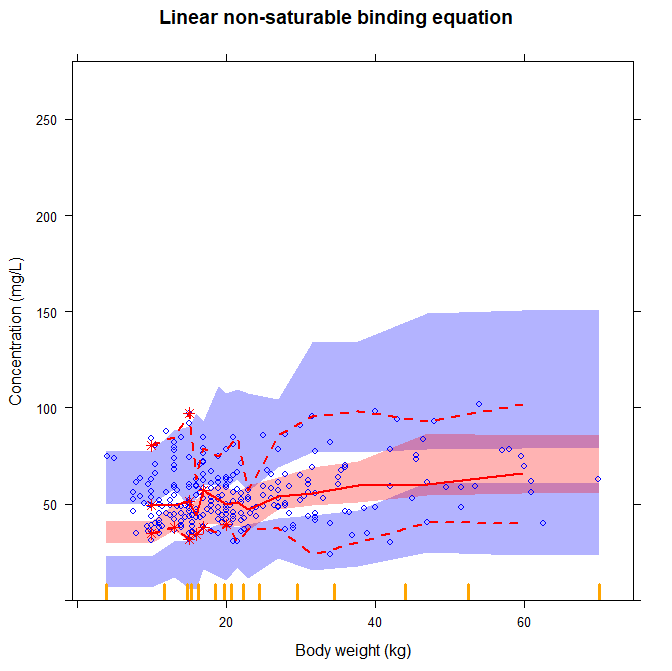 |
| --- | --- |

## Supplementary Figure. S3C

| 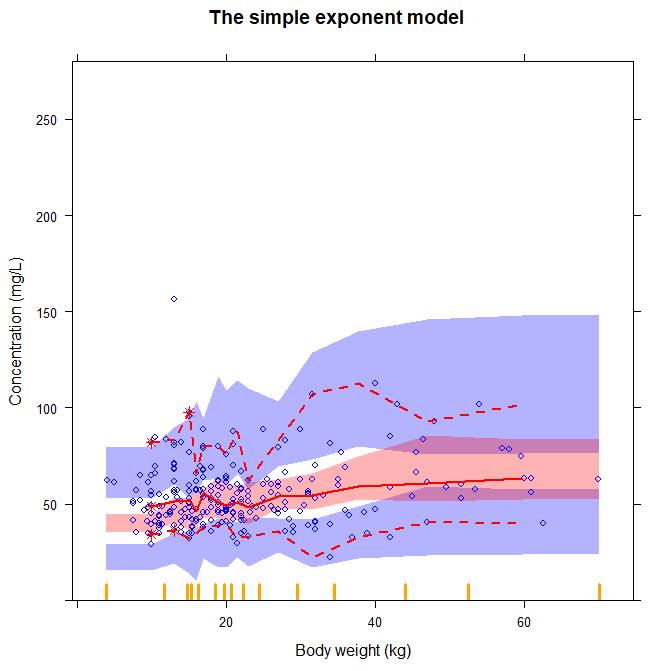 |  |
| --- | --- |

## Supplementary Figure. S4A


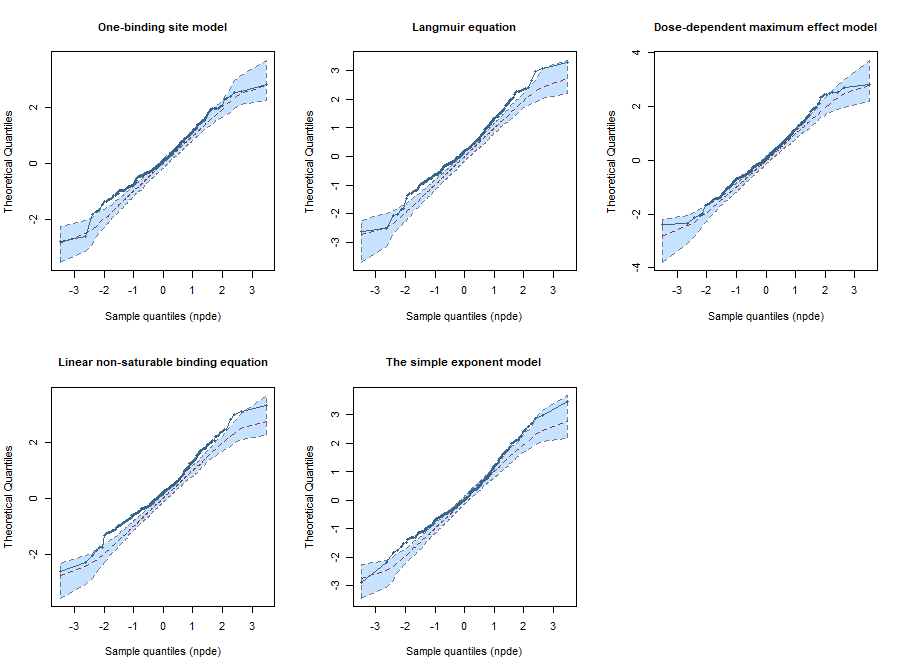


## Supplementary Figure. S4B


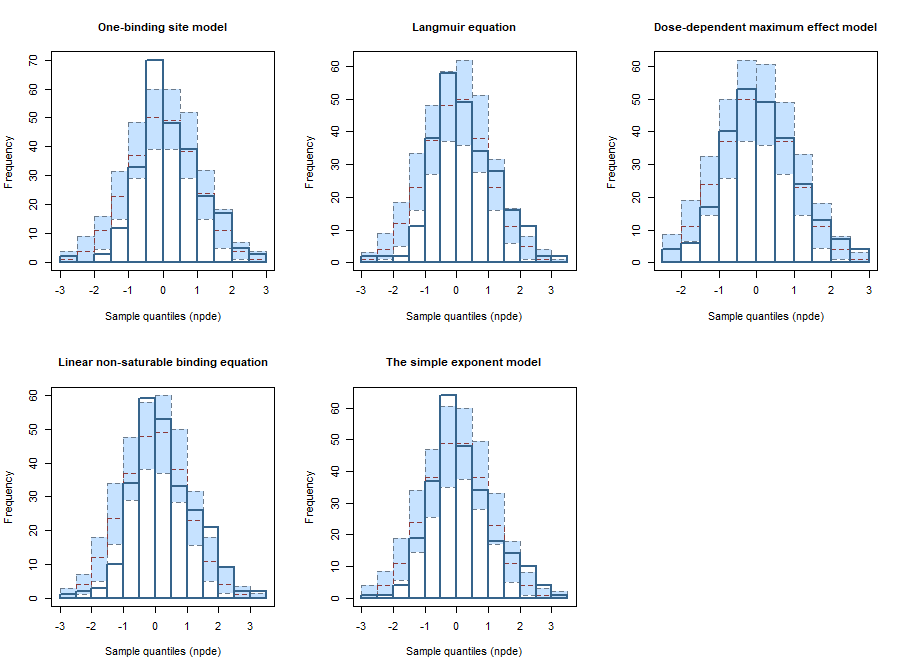


## Supplementary Figure. S4C

**
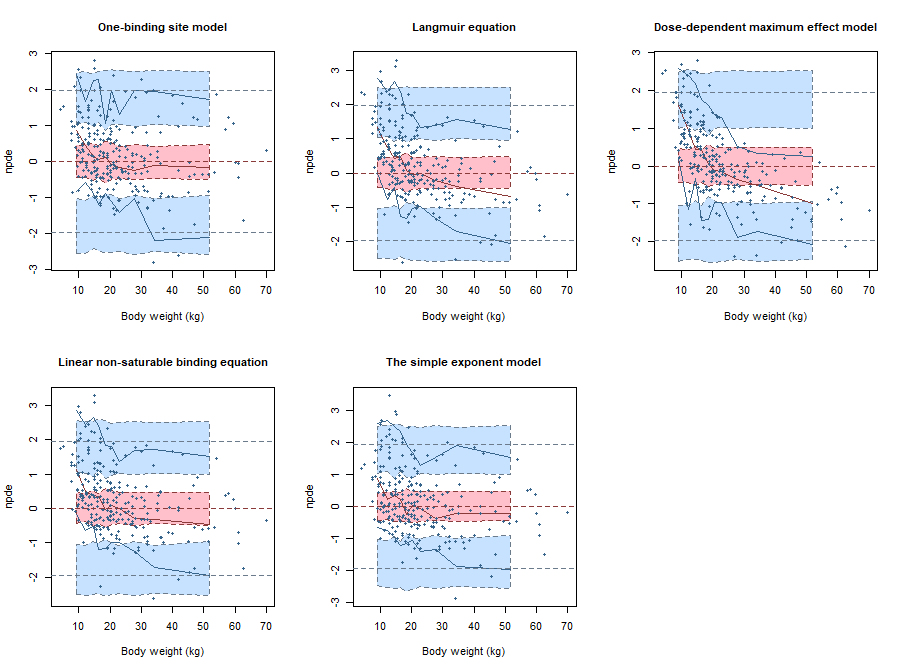
**

## Supplementary Figure. S4D


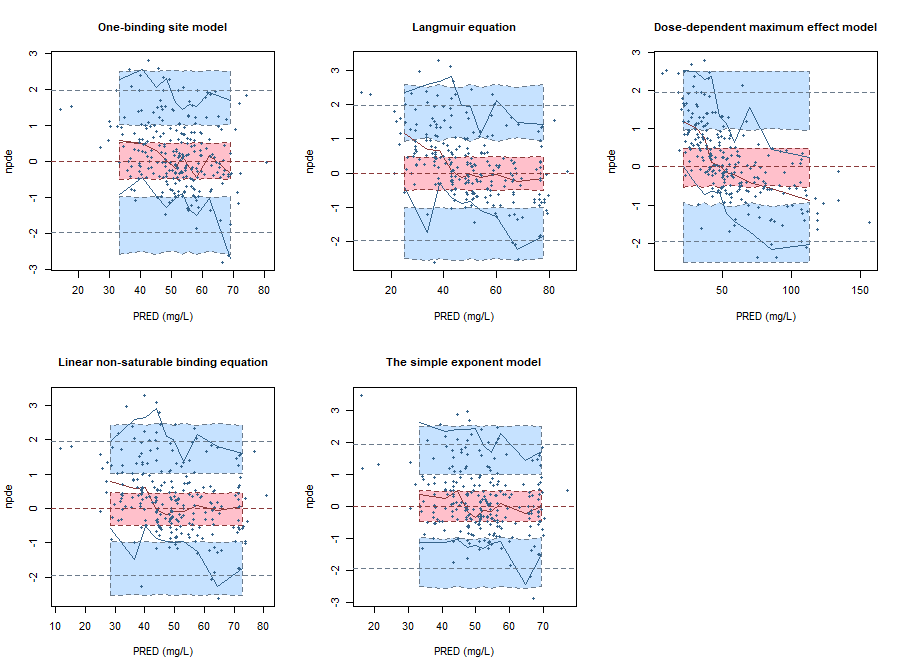


## Supplementary Figure. S5

| 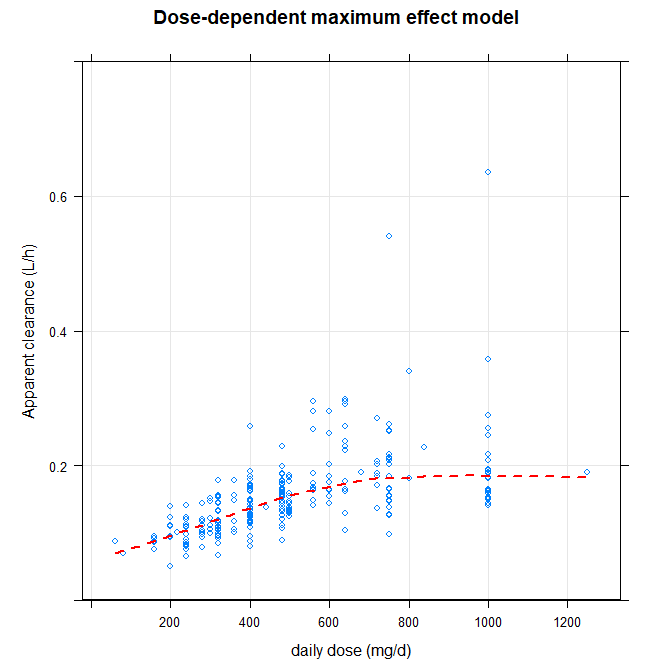  **A** | 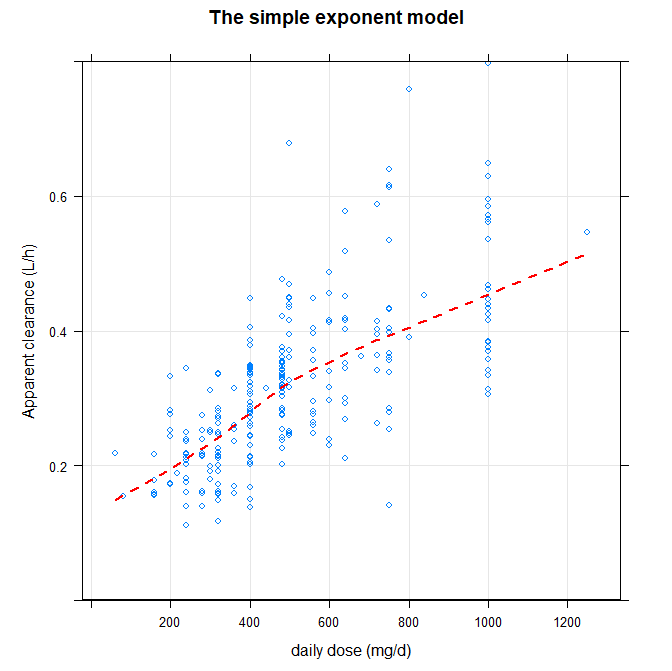  **B** |
| --- | --- |
