# Supplementary material for: Modeling the protein binding non-linearity in population pharmacokinetic model of valproic acid in children with epilepsy: a systematic evaluation study
Source: Front Pharmacol. 2023 Oct 6;14:1228641. doi: 10.3389/fphar.2023.1228641 (PMC10587682; doi:10.3389/fphar.2023.1228641)
Supplement: Supplementary file 4 [file DataSheet2.docx]

**Electronic Supplementary Material**

**Supplementary Text S1 Detailed Bayesian forecasting process**

Two general schemes for implementing Bayesian forecasting are conceivable when the maximum *a posteriori* Bayesian (MAPB) estimation method is implemented in a pharmacokinetic scenario. The “all data” method bases each forecast on the original *prior* updated with all concentration data for the patient as it becomes available. The “Sequential” method bases each forecast on a *prior* sequentially updated based on any previous individual concentrations and one new concentration only. The study by Mould *et al.* showed that the “all data” method has clear advantages over the “Sequential” method.

In our study, the individual prediction of the last observation was estimated based on one *prior* observation. Median IPE%, median absolute IPE%, and F_20_ and F_30_ of IPE% (IF_20_ and IF_30_, respectively) were used to evaluate predictability as *prior* information involved.

Reference: Mould DR, D'Haens G, Upton RN. Clinical Decision Support Tools: The Evolution of a Revolution. Clinical

pharmacology and therapeutics 2016; 99: 405-18.

Owen JS, Fiedler-Kelly J. Introduction to Population Pharmacokinetic/Pharmacodynamic Analysis with Nonlinear Mixed Effects

Models. John Wiley & Sons, 2014; 74.

**Supplementary Text S2 Detailed literature search process**

Inclusion criteria:

(1) Studied population: pediatric epilepsy patients administrated with valproic acid (VPA).

(2) Population Pharmacokinetics (popPK) study: modeling by parametric nonlinear mixed-effects approach.

(3) Language: English.

Exclusion criteria:

1. Reviews or methodological papers.
2. Model parameters were not available for external evaluation.
3. Datasets were overlapped or articles were duplicated.
4. PopPK studies included genetic polymorphisms as covariates.

Overview of the searching strategy:

Additionally publications identified from the reference lists of selected papers (n = 0)

727 articles identified by search strategy:

PubMed (n = 133); Web of Science (n = 259); EMbase (n = 335)

Studies excluded by reading title and/or abstract (n = 465)

Studies remained after database duplicates removed (n = 486)

Full-text articles retrieved for more detailed judgement (n = 21)

Studies excluded due to following reasons:

(1) Model parameters not available (n = 4)[1-4]

(2) Data overlapped or duplicate articles (n = 2)[5, 6]

(3) Review/methodology studies (n = 1)[7]

(4) Approaches other than nonlinear mixed-effects modelling (n = 1)[8]

(5) Non-English article (n = 1)[9]

(6) included genetic polymorphisms as covariates

(n = 2)[10, 11]

Studies involved for external evaluation (n = 10)

Searching terms used were:

“((‘valproic acid’ OR ‘valproate’) AND (‘population pharmacokinetic’ OR ‘nonlinear mixed effects’ OR ‘NONMEM’) AND (‘children’ OR ‘infants’ OR ‘pediatric’)) ”

^*^Studies excluded (n = 11) due to following reasons:

1. Reviews or methodological papers (n=1)

- The study conducted by Methaneethorn [7] was a systematic review.

1. Model parameters not available (n=4)

- The study by Botha *et al*. [1] missed the estimated value of V/*F*.
- The study by Yukawa *et al*. [2] missed the estimated value of V/*F*, K_a_.
- The study by Jankovic *et al*. [3] missed the estimated value of V/*F*, K_a_.
- The study by Goto *et al*. [4] missed the estimated value of V/*F*, K_a_.

1. Data or cohort overlapping (n=2)

- Two studies by Jankovic *et al*. [3, 5] had data overlapped; therefore, the latter one was chosen for the next assessment.
- Two studies by Jiang *et al*. [6, 12] had data overlapped, and the latter one was excluded since the former was conducted with a larger sample size.

1. Included genetic polymorphisms as covariates (n=2)

- Two studies by Mei *et al*. [10] and Xu *et al*. [11] included genetic polymorphisms as covariates, thus both of them were excluded due to that genotyping is not routinely performed in TDM of VPA.

1. Approaches other than nonlinear mixed effect modelling (n=1) or non-English article (n=1)

- The study by Sanchez-Alcaraz *et al*. [8] was excluded since it was not modeling by nonlinear mixed-effects approach.
- The study by Juarez-Olguin *et al*. [9] was excluded since it was not published in English.

**REFERENCE**

1 Botha JH, Gray AL, Miller R (1995) A model for estimating individualized valproate clearance values in children. Journal of clinical pharmacology 35 (10): 1020-1024 DOI 10.1002/j.1552-4604.1995.tb04020.x

2 Yukawa E, To H, Ohdo S, Higuchi S, Aoyama T (1997) Population-based investigation of valproic acid relative clearance using nonlinear mixed effects modeling: influence of drug-drug interaction and patient characteristics. Journal of clinical pharmacology 37 (12): 1160-1167 DOI 10.1002/j.1552-4604.1997.tb04301.x

3 Jankovic SM, Milovanovic JR, Jankovic S (2010) Factors influencing valproate pharmacokinetics in children and adults. International journal of clinical pharmacology and therapeutics 48 (11): 767-775 DOI 10.5414/cpp48767

4 Goto S, Seo T, Hagiwara T, Ueda K, Yamauchi T, Nagata S, Ando Y, Ishitsu T, Nakagawa K (2008) Potential relationships between transaminase abnormality and valproic acid clearance or serum carnitine concentrations in Japanese epileptic patients. The Journal of pharmacy and pharmacology 60 (2): 267-272 DOI 10.1211/jpp.60.2.0017

5 Jankovic SM, Milovanovic JR (2007) Pharmacokinetic modeling of valproate from clinical data in Serbian epileptic patients. Methods and findings in experimental and clinical pharmacology 29 (10): 673-679 DOI 10.1358/mf.2007.29.10.1116313

6 Jiang D, Bai X, Zhang Q, Lu W, Wang Y, Li L, Muller M (2009) Effects of CYP2C19 and CYP2C9 genotypes on pharmacokinetic variability of valproic acid in Chinese epileptic patients: nonlinear mixed-effect modeling. European journal of clinical pharmacology 65 (12): 1187-1193 DOI 10.1007/s00228-009-0712-x

7 Methaneethorn J (2018) A systematic review of population pharmacokinetics of valproic acid. British journal of clinical pharmacology 84 (5): 816-834 DOI 10.1111/bcp.13510

8 Sanchez-Alcaraz A, Quintana MB, Lopez E, Rodriguez I (1998) Valproic acid clearance in children with epilepsy. Journal of clinical pharmacy and therapeutics 23 (1): 31-34

9 Juarez-Olguin H, Lugo-Goytia G, Flores-Murrieta F, Ruiz-Garcia M, Lares Asseff I, Flores Perez J (2010) Effect of treatment and additional disease on pharmacokinetic of valproic acid in children with epilepsy. Revista de investigacion clinica; organo del Hospital de Enfermedades de la Nutricion 62 (6): 516-523

10 Mei S, Feng W, Zhu L, Li X, Yu Y, Yang W, Gao B, Wu X, Fang F, Zhao Z (2018) Effect of CYP2C19, UGT1A8, and UGT2B7 on valproic acid clearance in children with epilepsy: a population pharmacokinetic model. European journal of clinical pharmacology 74 (8): 1029-1036 DOI 10.1007/s00228-018-2440-6

11 Xu S, Chen Y, Zhao M, Guo Y, Wang Z, Zhao L (2018) Population pharmacokinetics of valproic acid in epileptic children: Effects of clinical and genetic factors. European journal of pharmaceutical sciences : official journal of the European Federation for Pharmaceutical Sciences 122: 170-178 DOI 10.1016/j.ejps.2018.06.033

12 Jiang D-c, Wang L, Wang Y-q, Li L, Lu W, Bai X-r (2007) Population pharmacokinetics of valproate in Chinese children with epilepsy. Acta pharmacologica Sinica 28 (10): 1677-1684 DOI 10.1111/j.1745-7254.2007.00704.x
