# Supplementary material for: Modeling the protein binding non-linearity in population pharmacokinetic model of valproic acid in children with epilepsy: a systematic evaluation study
Source: Front Pharmacol. 2023 Oct 6;14:1228641. doi: 10.3389/fphar.2023.1228641 (PMC10587682; doi:10.3389/fphar.2023.1228641)
Supplement: Supplementary file 5 [file DataSheet1.docx]

# Supporting information

Additional Supporting Information may be found in the online version of this article at the publisher’s web-site:

***Supplementary Text S1*** Detailed Bayesian forecasting process

***Supplementary Text S2*** Detailed literature search process

***Table S1*** Demographic characteristics for published population pharmacokinetic models

***Table S2*** Statistic test results of normalized prediction distribution errors (NPDE) diagnostics

***Table S3*** Parameter estimates of the base model and five protein binding models

***Figure S1A-E*** Prediction-corrected visual predictive check (pcVPC) plots of the published models. The median observed values per bin (red solid line), the 5^th^ and 95^th^ percentiles (red dashed lines) of the observations (blue circles), the 95 % confidence interval of the 5^th^ and 95^th^ percentiles (blue areas) and the confidence interval of the median (red area) are also shown.

***Figure S2 A-D*** Normalized prediction distribution error (NPDE) plots of the published models. A, quantile-quantile plot of the distribution of NPDE against theoretical distribution; B, histogram of the distribution of NPDE against theoretical distribution; C, NPDE versus body weight (kg); D, NPDE versus predicted concentrations.

***Figure S3A-C*** Prediction-corrected visual predictive check (pcVPC) plots of five protein binding models. The median observed values per bin (red solid line), the 5^th^ and 95^th^ percentiles (red dashed lines) of the observations (blue circles), the 95 % confidence interval of the 5^th^ and 95^th^ percentiles (blue areas), and the confidence interval of the median (red area) are shown.

***Figure S4 A-D*** Normalized prediction distribution error (NPDE) plots of five protein binding models. A, quantile–quantile plot of the distribution of the NPDE against theoretical distribution; B, histogram of the distribution of NPDE against theoretical distribution; C, NPDE versus body weight (kg); D, NPDE versus predicted concentrations.

***Figure S5*** Empirical Bayes estimates of valproic acid clearance (CL/*F*) in different daily dose levels based on established dose-dependent maximum effect model and the simple exponent model. The model-predicted typical CL/*F* (red dashed line) and the individual CL/*F* (blue circles) are shown. **A** for dose-dependent maximum effect model and **B** for the simple exponent model.
